# Predicting internal cell fluxes at sub-optimal growth

**DOI:** 10.1186/s12918-015-0153-3

**Published:** 2015-04-03

**Authors:** André Schultz, Amina A Qutub

**Affiliations:** Department of Bioengineering, Rice University, Main Street, Houston, 6500 USA

**Keywords:** Flux balance analysis, COBRA, BiGG, Genome-wide metabolic reconstructions, Constraint based models, Extreme pathways, Sub-optimal growth, Protein cost

## Abstract

**Background:**

Flux Balance Analysis (FBA) is a widely used tool to model metabolic behavior and cellular function. Applications of FBA span a breadth of research from synthetic engineering of biofuels to understanding evolutionary adaptations. FBA predicts metabolic reaction fluxes that optimize a given objective. This objective is generally defined for unicellular organisms by a theoretical reaction which simulates biomass production. FBA has been extremely successful at predicting in *E. coli* growth rates under different media and gene essentiality, amongst other things. In order to improve predictions, additional constraints are coupled with optimization of the biomass function. Studies have suggested, however, that unicellular organisms - like multicellular organisms - do not grow at optimal rates. To further improve FBA predictions, particularly of internal cell fluxes, new techniques to explore the sub-optimal solution space need to be developed.

**Results:**

We present an innovative FBA method called *corsoFBA* based on the optimization of protein cost at sub-optimal objective levels. Our method shows good agreement with experimental data of *E. coli* grown at different dilution rates. Maintaining the objective function close to its maximum value predicts metabolic states that closely resemble low dilution rates; while higher dilution rates can be mirrored by lowering the biomass production value. By using a modified version of Extreme Pathways, we are also able to quantify the energy production and overall protein cost for all possible pathways in the central carbon metabolism.

**Conclusion:**

Metabolic flux distributions at the optimal objective can be substantially different from the near-optimal distributions. Importantly, the behavior of *E. coli* central carbon metabolism can be better predicted by exploring the sub-optimal FBA solution space. The *corsoFBA* method presented here is able to predict the behavior of PEP Carboxylase, the glyoxylate shunt and the Entner-Doudoroff pathway at different glucose levels, a behavior not predicted by the minimization of metabolic steps and FBA alone. This technique can be used to better predict internal cell fluxes under different conditions, and *corsoFBA* will be of great help for the study of cells from multicellular organisms using Flux Balance Analysis.

**Electronic supplementary material:**

The online version of this article (doi:10.1186/s12918-015-0153-3) contains supplementary material, which is available to authorized users.

## Background

Genome-wide metabolic reconstructions provide a powerful platform for the analysis of metabolic pathways. Reconstructions for at least sixty-five different species spanning fifty-one different genera, including humans [1,2], are available today [3]. Such reconstructions have been used successfully in several fields of research, including metabolic engineering, evolutionary analysis and metabolic network properties [4]. The mathematical formulation of these reconstructions are called Constraint Based Models. These models are defined at the core by a sparse stoichiometric matrix, where each column represents a reaction, each row a metabolite, and each entry the corresponding stoichiometric coefficient.

A vast array of computational methods for the analysis of Constraint Based Models is available, and the number continues to grow [5,6]. Perhaps the most widely used of these methods is Flux Balance Analysis (FBA). FBA returns a flux distribution through the entire metabolism which optimizes (minimizes or maximizes) a given objective function or reaction, such as ATP or biomass production. This flux distribution satisfies a steady-state assumption, such that there is no net production or consumption of any metabolite. A pre-defined set of constraints, such as upper and lower bounds for each reaction and substrate availability, is also defined [7].

This technique is most commonly applied to metabolic reconstructions of unicellular organisms with the optimization of a theoretical Biomass reaction. This reaction consumes resources such as amino acids, nucleotides and ATP at the rate the given organism would need in order to grow and multiply [8,9]. FBA alone has predicted in *E. coli* the uptake and release rates of certain metabolites [10-12], cell growth rate under different environmental conditions [10,11] and gene essentiality [10] with great success. However, the prediction of internal cell fluxes remains a challenge [13], mainly due to four reasons:

1. *The FBA solution is not unique*. There are several fluxes within the model that are not well defined, but can exist within certain bounds while the objective function is being optimized [7]. These fluctuations then define an FBA solution space, rather than a single, unique output.
2. *Organisms might not be operating at maximum capacity* [14-18]. In this case, the objective function might not be fully optimized, but instead be in a near-optimal or sub-optimal state. Furthermore, Flux Variability Analysis shows that the FBA solution space increases drastically when considering a near-optimal to optimal state [18], exacerbating the possibility of multiple FBA solutions.
3. *The observed metabolic state is also not unique*. That is, a single flux distribution cannot explain all the flux states observed in the experiments, and variation occurs within the bacterial population. Wintermute et al. [19] proposes a *cloud theory* for metabolic regulation, where bacteria are allowed to fluctuate within a near-optimal solution space. The study also demonstrates that the variability of fluxes within this region matches the observed variability within the data.
4. *Thermodynamically infeasible loops can appear in the FBA output*. These are sets of pathways termed “*type III pathways*” [20], which are composed of internal reactions that can be combined to yield a steady-state with no net input or output. These cycles can clutter the FBA output, hindering any subsequent analysis [5].

To better predict internal fluxes, some studies have relied on additional thermodynamic constraints [21]. Several of these studies successfully reduce the FBA solution space, but still leave a variety of states that, although thermodynamically feasible, are physiologically improbable [22-27]. Other studies rely on the optimization of a thermodynamic cost, and may miss other key biological factors that govern metabolism [16,28-33].

Significant efforts have also been directed toward integrating omics data and FBA [34-38], but the generally low correlation between gene expression and the associated reaction flux makes this a challenging field [34]. Furthermore, the predictive power of these integrated omics-FBA models is limited, since experimental data is needed to predict the fluxes.

More recent studies have relied on the theory that *E. coli* chooses its pathways based on the minimization of protein cost [39,40] and number of metabolic steps [41]. Several successful methods based on these assumptions have been proposed, which include minimization of net metabolic flux [12], minimization of the number of steps in the metabolism [28,42,43] and enzymatic level constraints [44-49]. One alternative method utilizes Elementary Modes to pre-define the directionality of reactions, thus reducing the FBA solution space [50].

These methods have been very successful at improving predictions of growth rate, substrate usage and internal cell fluxes in unicellular organisms. Several studies have suggested, however, that unicellular organisms in reality grow at rates lower than those predicted by FBA alone [14-17]. Although some of the above-mentioned methods do predict growth rates lower than the standard FBA, they all rely on implementing additional constraints upon optimization of the objective function. Furthermore, although this approach has been successfully implemented in the study of cancer cells [49], this objective function will most likely not hold true for the analysis of healthy multicellular organisms, like mammals, as healthy cells in these systems have more complex objectives than to simply grow and multiply.

Additionally, these one-step optimization methods with additional constraints return a single flux distribution, and they are unable to explore the near-optimal solution space. This limitation is significant given a recent study has suggested the flux distribution of *E. coli* can vary freely within a near-optimal space [19]. Therefore, in order to further improve FBA predictions, especially as the field expands to include multicellular organisms, new techniques which explore the sub-optimal solution space need to be developed.

To address this need, we propose a two-step optimization FBA method for predicting internal fluxes, termed COst Reduced Sub-Optimal FBA (*corsoFBA*), which is suitable for sub-optimal objectives. Rather than imposing additional constraints when optimizing the objective function, we fix this objective at a predefined value. An estimated protein cost throughout the metabolism is then minimized in order to predict the internal cell fluxes. By varying the biomass production value, we are able to predict how changes in pathway usage depend on changes in other conditions, particularly glucose availability. Although this method is not well suited for predicting growth rates, we are able to predict metabolic flux distributions within a near-optimal solution space. Furthermore, by decomposing the model using an adapted version of the Extreme Pathway analysis, we are able to break down the energy production pathways and further understand the model behavior as glucose concentrations change. We validate our methods using *E. coli* as the model organism.

## Methods

### Cost calculation and implementation

Some of the methods most successful at predicting internal cell fluxes have been based on the theory that *E. coli* is subject to a protein cost constraint [44-49]. Five of such methods available in the literature, along with *corsoFBA*, are summarized in Table 1. Here we explore a similar principle, and calculate the protein cost of a reaction based on the net flux through the reaction (*J*), the enzyme molecular weight (*MW*), and a thermodynamic penalty for reversible reactions. The cost term used in *corsoFBA* is defined as:

$$J{\cdot}MW{\cdot}exp\left({\frac{\alpha\cdot\Delta_{r}G^{'o}}{R\cdot{}T}}\right) $$

**Table 1:** **Optimization methods with additional constraints**

**Method**	**Enzymatic cost**	**Constraint**
FBAwMC [44]	$\sum a_{i}J_{i}$	≤1
MOMENT [46]	$\sum g_{i}\cdot {MW}_{i}$	≤*C*
Tepper et al. [47]	$\sum M_{i} + \delta \cdot \sum g_{i}$	minimize
Shlomi et al. [49]	$\sum \frac {{MW}_{i}J_{i}}{k_{{cat}_{i}}}$	≤*C*
Holzhütter [28]	$\sum J_{i}^{+} + k_{{eq}_{i}}\cdot {J_{i}^{-}}$	minimize
corsoFBA	$\sum J_{i}{\cdot }{MW}_{i}{\cdot }exp\left ({\frac {\alpha \cdot \Delta _{r}{G^{\prime }}_{i}^{o}}{R\cdot {}T}}\right)$	minimize
**Variables**		
*a* - crowding coefficient		
*J* - flux through the reaction		
*g* - enzyme concentration		
*MW* - molecular weight of associated enzyme		
*M* - metabolite levels		
*δ* - model parameter		
*k*_*cat*_ - turnover number		
*k*_*eq*_ - thermodynamic equilibrium constant		
*C* - fraction of dry weight mass associated with proteins		

These methods calculate an overall cost through the metabolism by summing individual costs associated with each reaction. While previous methods have been used to constrain the FBA solution, *corsoFBA* optimizes this cost for any given growth rate.

Where *Δ*_*r*_*G*^′^^*o*^ is the standard Gibbs free energy of the given reaction, *R* is the ideal gas constant and *T* is the temperature. The molecular weight term is included to represent the amount of resources needed in order to produce a sufficient amount of enzymes to maintain the associated reaction flux. The thermodynamic penalty, applied only to reversible reactions, represents the cost associated with the change in the concentration of metabolites required for the reaction to flow in the desired direction. That is, a reaction with positive *Δ*_*r*_*G*^′^^*o*^ would require a large disparity in the concentration of the associated metabolites, so the cost term is multiplied by a positive number larger than one. On the other hand, if *Δ*_*r*_*G*^′^^*o*^ is negative, and the reaction is therefore favorable, the cost term is multiplied by a positive number between zero and one, given that little or no metabolic adjustment is necessary. It is worth noting that this thermodynamic cost does not directly represent a change in enzyme concentration, but rather an increase or decrease in the overall cost associated with the reaction based on its standard Gibbs free energy. Furthermore, this term is applied separately to forward and backward directions of reversible reactions, favoring the direction with negative standard Gibbs free energy. A similar thermodynamic cost, used without molecular weight considerations, has been applied by Holzhütter [28], weighing only the fluxes directed in the thermodynamically unfavorable direction.

Finally, the parameter *α* is chosen to be $0.02RT\frac {mol}{kcal}$, making the final thermodynamic cost simply:

$$exp\left(0.02\vphantom{\frac{mol}{kcal}}\right.\cdot \Delta_{r}G'^{o}\left.\frac{mol}{kcal}\right). $$

This value is chosen in order to balance the contribution of molecular weight and thermodynamic penalty, yielding a prediction considering both costs (Additional file 1: Figure S1). Without this parameter, due to the exponential nature of the thermodynamic penalty, this term would quickly approach extremely large numbers (for positive values of *Δ*_*r*_*G*^′^^*o*^) or zero (for negative values of *Δ*_*r*_*G*^′^^*o*^), and only the thermodynamic cost would essentially be considered. In that case, reaction directionality would essentially be predetermined based solely on the standard Gibbs free energy, which could be problematic.

The Gibbs free energy of reactions was estimated from the MetaCyc database [51] as the change in Gibbs energy of formation between reaction compounds. Molecular weight values were obtained for 506 of the 523 (96.75%) enzyme catalyzed reactions in the *E. coli* iJR904 reconstruction [52]. These values were extracted from the BRENDA database [53] whenever available. If *E. coli* measurements were not available, we extracted the values for the organism most closely related to *E. coli*. A small number of molecular weights was estimated from the EcoCyc database [54], and the remaining 17 values were defined as the median of the calculated values. Additional information concerning the standard Gibbs free energy of reactions, protein molecular weights and protein cost calculations can be found in the supplemental information (Additional file 2, Additional file 3 and Additional file 4: Table S1).

Since the protein cost term scales linearly with the flux through the associated reaction, we incorporated the molecular weight and thermodynamic cost directly into the model. All enzyme associated reactions were split into forward and backward parts, and the cost was added as a produced metabolite:

$$ \begin{aligned} &\mathbf{Initial\,\,Reaction}\\ &rxn: A+B \iff C+D\\ &\mathbf{Reactions\,\,with\,\,cost}\\ &{rxn}_{f}: A+B \Rightarrow C+D+{MW}_{rxn}{\cdot}exp\left({\frac{\alpha\cdot\Delta_{r}{G^{\prime}}_{rxn}^{o}}{R\cdot{}T}}\right)\\ &{rxn}_{b}: C+D \Rightarrow A+B+{MW}_{rxn}{\cdot}exp\left({\frac{-\alpha\cdot\Delta_{r}{G^{\prime}}_{rxn}^{o}}{R\cdot{}T}}\right)\\ \end{aligned} $$

A reaction which consumes this produced metabolite was then added to the model. While every original reaction in the model produces this cost at a rate relative to its absolute flux, this added reaction becomes the only one to consume this cost. Minimizing the flux through this reaction will then return the flux distribution with the lowest enzyme associated cost to achieve the predefined biomass production value. Reactions with no associated enzyme were given a cost of zero.

These methods were applied to the *E. coli* iJR904 reconstruction [52]. During all simulations the glucose uptake bound was set to an arbitrary value. Uptake bounds for *CO*_2_, *Fe*_2_, *H*_2_O, H, Na, *NH*_4_, *O*_2_, Phosphate, and *SO*_4_ were set to arbitrarily large values, much larger than the glucose uptake bound, and were considered to be present in excess. All exchange reactions release bounds were set to arbitrarily large values except for glucose, formate and *O*_2_, which were set to zero. Predicted flux values were then normalized by either the flux of glucose to glucose-6-phosphate or overall flux through enzyme associated reactions.

It is worth noting that this protein cost term does not account for the enzyme turnover rates or substrate affinity. We have opted for this cost term since the kinetic parameters of enzymes are extremely difficult to obtain experimentally, and vary greatly based on pH and temperature values. Furthermore, the simplification of a constant turnover rate and substrate affinity for all enzymes has been shown to yield significant results [39].

### Fundamental pathways analysis

Different methods have been proposed to characterize and decompose the FBA solutions space [5,55], including Elementary Modes [56,57], Extreme Pathways [20] and Minimal Generators [58]. However, these tools have mostly been applied to small networks. The number of pathways obtained for each of these methods, as well as the computational cost of these analyses, increase exponentially with the size of the reconstruction [5,59]. With large-scale metabolic reconstructions, these decompositions quickly become impractical tools. Here we propose a modified version of Extreme Pathways which yields a significantly lower number of pathways during the decomposition. Each of these pathways is associated with a protein cost and an ATP production potential, which helps elucidate the model behavior as the value of the objective function varies.

We find that this large number of pathways stems partially from the need to balance each pathway to a steady-state, such that no metabolite has a net production or consumption rate. Certain metabolites, however, known as currency metabolites, are responsible for basic cell functions and occur in numerous reactions in the model. Standard reactions involving these metabolites, such as ATP hydrolysis, can be obtained through different combinations of other model reactions. These combinations, or loops, can then be used multiple times with the same purpose, increasing the number of pathways which have essentially the same core reactions.

**Figure 1:**
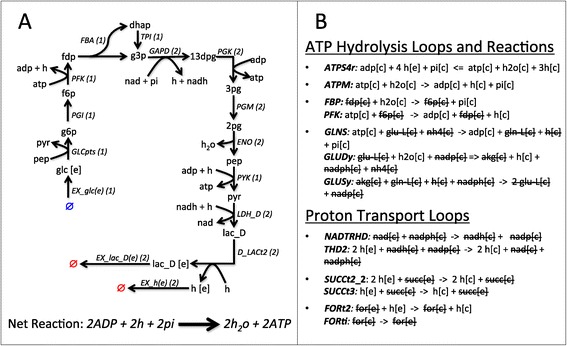
**Metabolic loops increase pathway multiplicity. (A)** Pathway converting 1 mol of glucose to 2 mols of lactate and producing 2 mols of ATP. Reaction fluxes are represented in parentheses. Extracellular metabolites are defined by [e]. All other metabolites are in the cytosolic space. **(B)** Network loops and reactions that can hydrolyze ATP and transport protons across the cell membrane. These reactions and loops can be used to balance the reactions in **(A)**, yielding multiple balanced pathways with the same set of backbone reactions

This phenomenon can be illustrated by Extreme Pathways analysis applied to the pathway and set of reactions presented in Figure 1. The pathway depicted in Figure 1A is the production of energy through the conversion of glucose into lactate, which produces two mols of ATP for every mol of glucose. Reaction names are extracted from the *E. coli* reconstruction by Orth et al. [60]. Figure 1B illustrates loops and reactions present in the same reconstruction that hydrolyze ATP, any of which can be combined with the reactions in Figure 1A to produce a pathway satisfying the ATP steady-state condition. Furthermore, *ATPS4r* also introduces a proton imbalance when hydrolyzing ATP. This imbalance can then be corrected by any of the proton transport loops also presented in Figure 1B. The combination of this pathway with the given loops then give us multiple Extreme Pathways with essentially the same core reactions; and they represent the same phenomenon - the conversion of glucose to lactate through glycolysis.

In order to extract only the core reactions in each pathway, we then remove a selected set of currency metabolites from the reconstruction. This removal reduces the number of pathways we obtain from Extreme Pathway analysis. This metabolite removal was performed under certain considerations:

1. *Metabolites removed should be completely deleted from all reactions and compartments in the model*. Removing a metabolite from one reaction and not others could block certain reactions, since fewer options to balance that metabolite would be present in the reconstruction.
2. *No reaction should become a sink or source of metabolites*. Allowing reactions to become sinks or sources would introduce exchange reactions for certain metabolites, affecting the model decomposition.
3. *Reactions removed from the reconstruction should not contain any metabolite which has not been removed from the reconstruction*. The removal of these reactions would remove any pathway which uses these reactions to balance the metabolite still in the model.

While we should be aware of these considerations when modifying the model, breaking any of these specifications should not invalidate the Fundamental Pathway analysis. The model decomposition could still be performed, but some limitations might have to be considered. We have illustrated this Fundamental Pathways method using the *E. coli* model by Orth et al. [60], and have demonstrated that the original pathways can be recovered by adding back the identified loops. This analysis can be found in the supplemental information (Additional file 5 and Additional file 6: Table S2).

While we obtain fewer pathways in this tailored reconstruction, each set of reactions is associated with an imbalance in the original model. For example, instead of multiple Extreme Pathways, the set of reactions in Figure 1A would yield a single fundamental pathway after the removal of currency metabolites from the reconstruction. These reactions alone, however, would yield ATP, ADP, phosphate, proton and water imbalances in the original model. In the Fundamental Pathways analysis, each resulting fundamental pathway is then associated with this imbalance, which can be used to further analyze each pathway. In this study, this imbalance is used to analyze the ATP production capacity of each pathway obtained.

## Results and discussion

### Comparison between different cost functions

Cost-optimal flux simulations were performed using four different types of cost: (1) molecular weights alone, (2) thermodynamic penalties alone, (3) the previously mentioned combination of molecular weights and thermodynamic penalties and (4) uniform costs. The use of uniform costs gives a simple minimization of the overall flux through the enzyme associated reactions. This minimization strategy is a modified version of a widely regarded two-step optimization method called *pFBA* [12]. *pFBA* has been previously used to predict unique flux distributions at the predicted FBA optima.

Simulations comparing these four costs have been performed for growth rates ranging from 50 to 100% of the predicted optima. Flux distributions were normalized by the flux of glucose to glucose-6-phosphate, and results for several central carbon metabolism reactions can be found in the supplemental information (Additional file 7: Figure S2). While simulations using molecular weights, thermodynamic costs, or a combination of both present considerably different flux distributions at different sub-optimal values, simulations using uniform costs quickly converge to a unique flux distribution as the objective value is lowered.

The same set of simulations was also compared to several data points from two Metabolic Flux Analysis (MFA) experiments: Ishii et al. [61] and Yao et al. [62]. Correlation and sum of squared error between all simulated and experimental flux distributions for several central carbon metabolism reactions were calculated. These results are presented in the supplemental information (Additional file 8: Figure S3). A similar method to that used by Holzhütter [28] was also included in Additional file 8: Figure S3. While *pFBA* simulations yield the highest correlation values in the suboptimal space when compared to the Ishii et al. dataset, these same simulations also yield the highest sum of squared error. Furthermore, when the objective function was lowered from 100% down to 65% of optima, *corsoFBA* yields the lowest sum of squared error for all data points except one (Ishii et al. with dilution of 0.7h^-1^), and the highest correlation values for the higher dilution rates in the Yao et al. data (dilution = 0.4, 0.6 and 0.7h^-1^).

### Comparison between metabolic flux analysis and simulated fluxes

**Figure 2:**
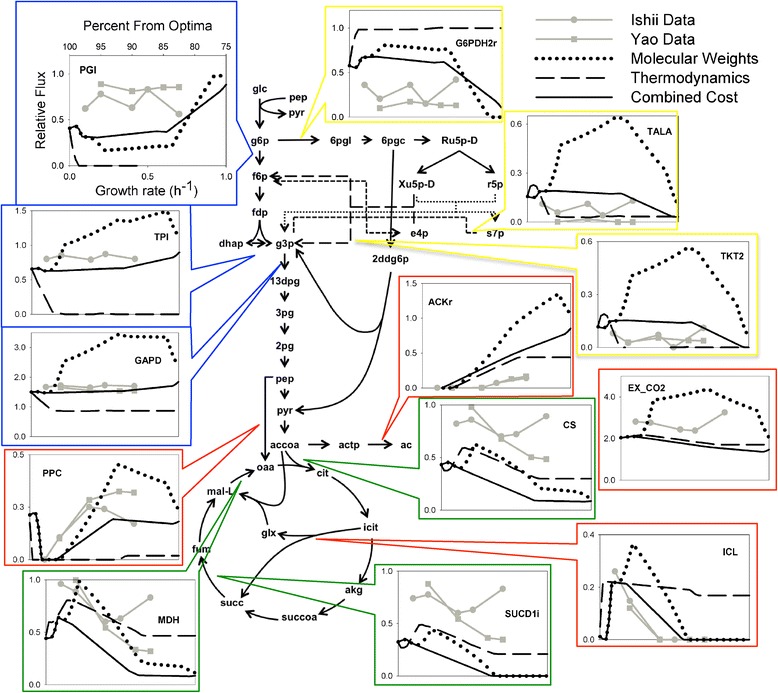
**Comparison between simulations and MFA experiments.** Comparison between selected simulated fluxes using molecular weights, thermodynamic penalties, and a combination of the two costs, and Metabolic Flux Analysis (MFA) data. Fluxes are normalized by glucose to glucose-6-phosphate conversion rates. X-axes values are kept constant for all plots. Reaction names are taken from the *Ecoli iJR904* model [52]. Yellow boxes indicate Pentose Phosphate Pathway (PPP), blue indicates glycolysis, green indicates TCA cycle and red indicates other reactions

In order to visualize the trend of how different fluxes change in the sub-optimal space, simulations utilizing molecular weight costs, thermodynamic costs and a combination of both, normalized again by the flux of glucose to glucose-6-phosphate, were plotted alongside the experimental values from the two MFA experiments. Fluxes were plotted starting at 100% down to 75% of the predicted optima, and the results for several central carbon metabolism reactions are presented in Figure 2. The threshold of 75% was chosen in order to match the trend observed in our simulations to the experimental data [61,62].

As the objective function value decreases, *corsoFBA* flux distributions show close agreement with MFA data for higher growth rates (Figure 2), particularly for simulations considering both thermodynamic and molecular weight costs. Fluxes through the Pentose Phosphate Pathway (PPP) and Glycolysis remain relatively constant, while the flux through the TCA cycle gradually decreases. The decrease in TCA cycle usage leads to higher levels of acetate release. Quantitatively, the fluxes through glycolysis and the TCA cycle are slightly under-predicted by our simulations, while the flux through the PPP is generally over-predicted. Similar patterns have been observed in other FBA method approaches [33,45,48], and this may arise due to the optimization of the biomass function alone. The optimization of ATP production alongside biomass has been shown to yield better predictions [16,63]. ATP production optimization could also lead to higher fluxes through the TCA cycle and lower PPP fluxes. It is also worth noting that, although fluxes through the TCA cycle are under-predicted according to Figure 2, MFA experimental results can be inconsistent, and our predicted TCA fluxes show good agreement with two other MFA studies [64,65].

These simulations mirror a behavior known as *overflow metabolism* [66], where *E. coli*, at high growth rates, moves away from the full oxidation of glucose through the TCA cycle and uses less ATP efficient pathways, releasing acetate instead of CO _2_. Our simulations support the hypothesis that this behavior stems from a tradeoff between enzyme cost and energy yield [40]. That is, when more glucose is available, *E. coli* uses a higher flux through pathways that are less enzymatically costly, but which produce fewer ATP per mmol of glucose.

Predicted fluxes using molecular weights only and a combined cost also found excellent agreement with the experimental values in the reactions PPC (PEP Carboxylase) and ICL (Isocitrate Lyase)[61,62]. Both experiments show a decrease in ICL flux and an increase in PPC flux, from near-optimal conditions to a growth rate of 0.5 h^-1^. This result is further supported by the experimental results from Nanchen et al. [67], that found a lower flux through ICL during a growth rate of approximately 0.05 h^-1^ when compared to a growth rate of 0.1 h^-1^, suggesting a transient response through this pathway as glucose concentration increases. The same transient response was found in our simulations. These results suggest that *E. coli* utilizes these anaplerotic reactions (reactions responsible for forming intermediates for metabolic pathways) to relieve enzymatic cost, and that considering these costs during FBA may increase internal flux predictions when comparing to the minimization of metabolic steps alone.

Simulation results also suggest that the optimization of the biomass function may yield fluxes that are fundamentally different from those at near-optimal conditions. At optimal growth, there is no predicted flux through the glyoxylate shunt, considerably lower fluxes through the TCA cycle and PPP, and a flux through the PPC reaction not present at near-optimal conditions. Although comparisons between experimental data and simulated flux distributions show that the highest correlation and lowest error are found at optimal growth, our simulations also indicate that the implementation of these costs yield comparable results in the sub-optimal space (Additional file 8: Figure S3). Moreover, the suboptimal *corsoFBA* approach can better predict fluxes through certain reactions, such as PPC and ICL. While previous studies have shown that the FBA solution space increases drastically when considering the objective function at near-optimal to optimal values [18], here we show that the optimization of the enzymatic cost at near-optimal conditions yields results that are more consistent with experimental data for certain reactions. This result strongly suggests that exploring the FBA solution space at near-optimal to optimal objective values may increase the predictive accuracy of internal cell fluxes.

The near optimal solution space was also analyzed for knockout strains. Flux distributions for six *E. coli* knockout strains reported by Ishii et al. [61] were compared to cost-optimal simulations using the combined cost function. Results show that cost-optimal flux distribution from 95% to optimal yield similar or better predictions according to both correlation and sum of square error (Additional file 9: Figure S4). Predictions using uniform costs quickly diverge from experimental data. These simulations further support the “cloud theory” proposed by Wintermute et al. [19], and suggest that the experimental data can also be in good agreement with near-optimal flux distributions.

### Comparison between gene expression data and simulated fluxes

**Figure 3:**
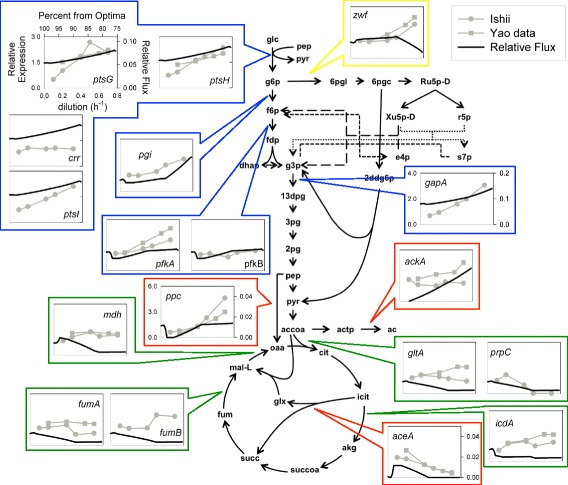
**Comparison between simulations and gene expression data.** Comparison between selected simulated fluxes, using a combination of molecular weights and thermodynamic penalties, and associated gene expression. Plot axes are the same as the ones defined in the *ptsG* plot unless otherwise specified. Multiple genes associated with the same reaction are included in the same box. Reaction names are taken from the *E. coli iJR904* reconstruction [52]. Yellow boxes indicate Pentose Phosphate Pathway, blue indicates glycolysis, green indicates TCA cycle and red indicates other reactions

To further validate this analysis, simulations using the combined cost function were also compared to gene expression values reported in the MFA studies [61,62]. While the overall correlation between reaction flux and gene expression is moderate at best [34], increased expression of all genes participating in a particular pathway can be considered a good indication of increased flux [68]. Simulations were performed under the same conditions as before, utilizing the combination of molecular weights and thermodynamic penalty, but this time the flux distribution was normalized by the overall flux through the enzyme associated reactions. Comparisons between simulated values and gene expression data are shown in Figure 3.

Although gene expression data can also be inconsistent between experiments, and some reactions are associated with multiple genes, this comparison further supports the qualitative results presented in the previous section. When normalizing the predicted fluxes by the overall flux through the enzyme associated reactions, an increased relative uptake of glucose is observed as the objective value decreases. As a result, an increased relative flux through glycolysis is also predicted. Furthermore, the relative flux through the TCA cycle still decreases as the growth rate increases. The increase in relative glucose uptake and flux through glycolysis, as well as the decreased flux through the TCA cycle, are supported by the relative gene expression associated with these pathways [68].

Simulated values also find good agreement with fluxes through the Entner-Doudoroff (ED) pathway. Genes *edd* and *eda*, associated with this pathway, have long been known to exist in *E. coli* [69], but the activity of their associated reactions has been observed mainly under growth on gluconate, glucuronate, and methyl-beta-D-glucuronide; phosphate limitation; and carbon starvation [70]. Due to this fact, these reactions are generally not included in MFA experiment networks. In contrast with Murray et al., our simulations using molecular weight and combined costs predict the use of the ED pathway under excess glucose conditions, not starvation (Additional file 7: Figure S2). These predictions are supported both by the genetic data presented by the MFA studies [61,62], which show an increase in *edd* and *eda* expression at high glucose concentrations, and MFA experimental results by Harcombe et al. [71], where fluxes through the ED pathway were measured in bacteria growing at growth rates above 1 h^-1^.

### Fundamental pathways analysis

**Figure 4:**
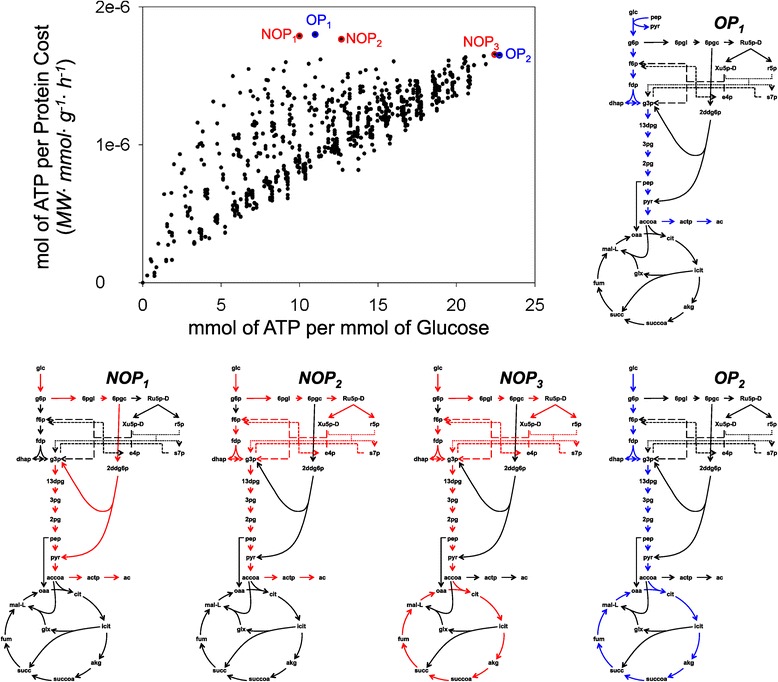
**Fundamental pathway analysis.** ATP production potential of energy producing fundamental pathways, compared to the associated protein cost of each mmol of ATP. Optimal (OP) and Near Optimal (NOP) Pathways are highlighted in the scatter plot. OPs are plotted in blue and NOPs in red in the central carbon metabolism diagrams

To better understand the transition between metabolic states as the value of the objective function is decreased, the energy producing pathways of the *E. coli iJR904* reconstruction were decomposed using the Fundamental Pathways analysis described in the Methods section. Details on how these reactions were calculated can be found in the supplemental information (Additional file 5 and Additional file 6: Table S2). Briefly, starting with the ATP imbalance associated with each fundamental pathway, the total ATP potential of each pathway was estimated based on the imbalance of other energy generating metabolites, such as NADH and Ubiquinol-8. The lowest cost of converting these metabolites into ATP was then added to the total cost associated with the pathway. The final ATP production by enzymatic cost was then compared to the potential ATP production by mmol of glucose (Figure 4).

In accordance with general *E. coli* metabolism knowledge, the fundamental pathway analysis found two optimal pathways (OP), according to ATP production by both mmol of glucose and protein cost. The most energy productive pathway is the full oxidation of glucose through the TCA cycle (*OP*_2_). The most cost efficient pathway was found to be the production of acetate through glycolysis (*OP*_1_). Although this pathway potentially produces less than half the amount of ATP as *OP*_2_, the total enzymatic cost per mmol of ATP is lower. Also in agreement with general knowledge, this analysis predicted the release of acetate to be more efficient than the release of both lactate and ethanol.

In the simulations presented in previous sections, a gradual transition from *OP*_2_ to *OP*_1_ is observed as the objective function value decreases. This trend also supports the idea that *overflow metabolism* takes place in order to alleviate enzymatic cost. Under low glucose conditions, *E. coli* would need to extract from glucose the largest possible amount of energy in order to sustain growth. As more glucose becomes available, however, this bacteria can afford to consume more substrate and produce energy through cheaper pathways.

One interesting observation from this analysis is the existence of Near Optimal Pathways (NOP), which combine the previously described optimal pathways with the PPP or ED pathways. *NOP*_2_ and *NOP*_3_ both produce more ATP per mmol of glucose than *OP*_1_ but less than *OP*_2_, while remaining less cost efficient than *OP*_1_ but slightly more so than *OP*_2_. *NOP*_2_ and *NOP*_3_ reduce the flux through upper glycolysis by partially re-routing the flux through the PPP, and they exhibit no flux through Phosphoglucose isomerase (pgi). A third Near Optimal Pathway, termed *NOP*_1_, skips the upper glycolysis through the ED pathway. This pathway is both less cost and energy effective than *OP*_1_.

The use of these near optimal pathways could explain the inconsistent PPP fluxes reported by the MFA experiments considered here. A short recovery in the simulated PPP flux near a growth rate of 0.65 h ^−1^ (Figure 2) demonstrates that this pathway is a viable option at certain dilution rates, due to its coupling with the PPP and biomass production. It has also been demonstrated that *Δ*pfkA deficient *E. coli* strains reduce flux through Phosphofructokinase 1 by diverting fluxes through the PPP [72], hence using *NOP*_2_ and *NOP*_3_ instead of *OP*_1_ or *OP*_2_ when the cost through upper glycolysis is increased.

This Fundamental Pathway analysis elucidates how we would expect the model to behave in terms of energy generation as we optimize the protein cost at sub-optimal states. Based on this analysis, in order to fulfill its catabolic needs, the model transitions from *OP*_2_ to *OP*_1_ as we move away from optimal growth. Although this is in fact the general trend we observe, anaplerotic needs also need to be considered. These needs are addressed most efficiently by reactions not in *OP*_1_ or *OP*_2_, such as PEP carboxylase and the glyoxylate shunt. Interestingly, the model also predicts the use of the ED pathway with anaplerotic purposes at high growth rates. While this pathway has been studied mostly for its catabolic activity in *Z. mobilis* and several *Pseudomonas* species, simulations here predict this pathway to be the cheapest way to get glucose shuttled to the TCA cycle for the production of building blocks, while giving up little efficiency in energy pathways through *NOP*_1_.

## Conclusion

Here we present *corsoFBA*, a method for analyzing and predicting metabolic flux distributions at sub-optimal states based on protein and thermodynamic cost minimization. *CorsoFBA* demonstrates that the optimization of protein cost at near-optimal states can produce significantly different results from those at the optimal objective. Furthermore, although correlation and error calculations indicate better predictions at the optimal solution, protein-optimal results at sub-optimal objectives show better agreement with MFA experiments and gene expression profiles for anaplerotic reactions. These results suggest it is important to explore the sub-optimal FBA space in order to better predict internal cell fluxes. Although the method described here is not suited for predicting growth rates, it provides a platform for analyzing internal cell fluxes in the sub-optimal space.

We believe *corsoFBA* will be particularly useful given recent studies have suggested *E. coli* can exist freely within a near-optimal space [19]. We also believe this method can be useful in predicting fluxes in healthy, multicellular organisms, which have more complex objectives than the production of biomass. Future studies are merited to explore whether the predictive power of methods which optimize the objective function under enzymatic level constraints, such as MOMENT [46] and FBAwMC [44], would also benefit from exploring the sub-optimal solution space. Our results suggest that significant conclusions could be drawn by adapting these methods to optimize the enzymatic cost they implement in a near-optimal space, rather than using these simply as a model constraint.

## Additional files

Additional file 1
**Figure S1.** Sensitivity analysis of parameter *α*. Model simulations ranging from 100 to 75% of optima with varying values of *α*. Simulations are performed using both thermodynamic costs only and combined costs.

Additional file 2
**Gibbs free energy change distribution.** File showing plot and distribution of the Gibbs free energy change of reactions considered.

Additional file 3
**Molecular weight and cost calculation.** Details on how Molecular weight values were obtained and final costs calculated. Molecular weights and final costs distribution are also plotted.

Additional file 4
**Table S1.** Thermodynamic, molecular weight and final cost values. Table includes all values used during simulations: Gibbs energy of formation for all metabolites, calculated Gibbs energy change for all reactions based on metabolite energy of formation and molecular weights obtained from databases.

Additional file 5
**Fundamental pathway analysis.** Example demonstrating the recovery of *Extreme Pathways* by adding reactions loops back into the *Fundamental Pathways*. Details on how the fundamental pathway analysis presented in the manuscript was performed are also available.

Additional file 6
**Table S2.** Model decomposition. *Extreme Pathways* and *Fundamental Pathways* calculated both for the analysis presented in the manuscript and the pathway recovery analysis in Additional file 5.

Additional file 7
**Figure S2.** Cost values comparison. Cost-optimal simulations using molecular weights alone, thermodynamic penalties alone, a combination of the two and uniform costs are presented for major central carbon metabolism reactions in the range of 100 to 50% of maximal growth.

Additional file 8
**Figure S3.** Error and correlation calculations. Correlation and sum of squared error are calculated between all simulated and experimental flux distributions for major central carbon metabolism reactions.

Additional file 9
**Figure S4.** Error and correlation calculations for knockout strains. Correlation and sum of squared error are calculated between MFA experimental data and simulated flux distributions using the combined cost function for six knockout strains.

## Abbreviations

TCA: Tricarboxylic acid
FBA: Flux balance analysis
PPP: Pentose phosphate pathway
MFA: Metabolic flux analysis
ED: Entner-Doudoroff
glc: glucose
g6p: glucose-6-phosphate
f6p: fructose-6-phosphate
fdp: fructose-1,6-biphosphate
dhap: Dihydroxyacetone phosphate
g3p: Glyceraldehyde 3-phosphate
13dpg: 3-Phospho-D-glyceroyl phosphate
3pg: 3-Phospho-D-glycerate
2pg: D-Glycerate 2-phosphate
pep: Phosphoenolpyruvate
pyr: Pyruvate
accoa: Acetyl-CoA
actp: Acetyl phosphate
ac: Acetate
cit: Citrate
icit: Isocitrate
akg: 2-Oxoglutarate
succoa: Succinyl-CoA
succ: Succinate
fum: Fumarate
mal-L: L-Malate
oaa: Oxaloacetate
6pgl: 6-phospho-D-glucono-1,5-lactone
6pgc: 6-Phospho-D-gluconate
ru5p-D: D-Ribulose 5-phosphate
r5p: alpha-D-Ribose 5-phosphate
xu5p-D: D-Xylulose 5-phosphate
s7p: Sedoheptulose 7-phosphate
e4p: D-Erythrose 4-phosphate
2ddg6p: 2-Dehydro-3-deoxy-D-gluconate 6-phosphate
