# Supplementary material for: Predicting internal cell fluxes at sub-optimal growth
Source: BMC Syst Biol. 2015 Apr 3;9:18. doi: 10.1186/s12918-015-0153-3 (PMC4397736; doi:10.1186/s12918-015-0153-3)
Supplement: Additional file 1 — Figure S1. Sensitivity analysis of parameter α. Model simulations ranging from 100 to 75% of optima with varying values of α. Simulations are performed using both thermodynamic costs only and combined costs. [file 12918_2015_153_MOESM1_ESM.pdf]

Thermodynamic Costs Only

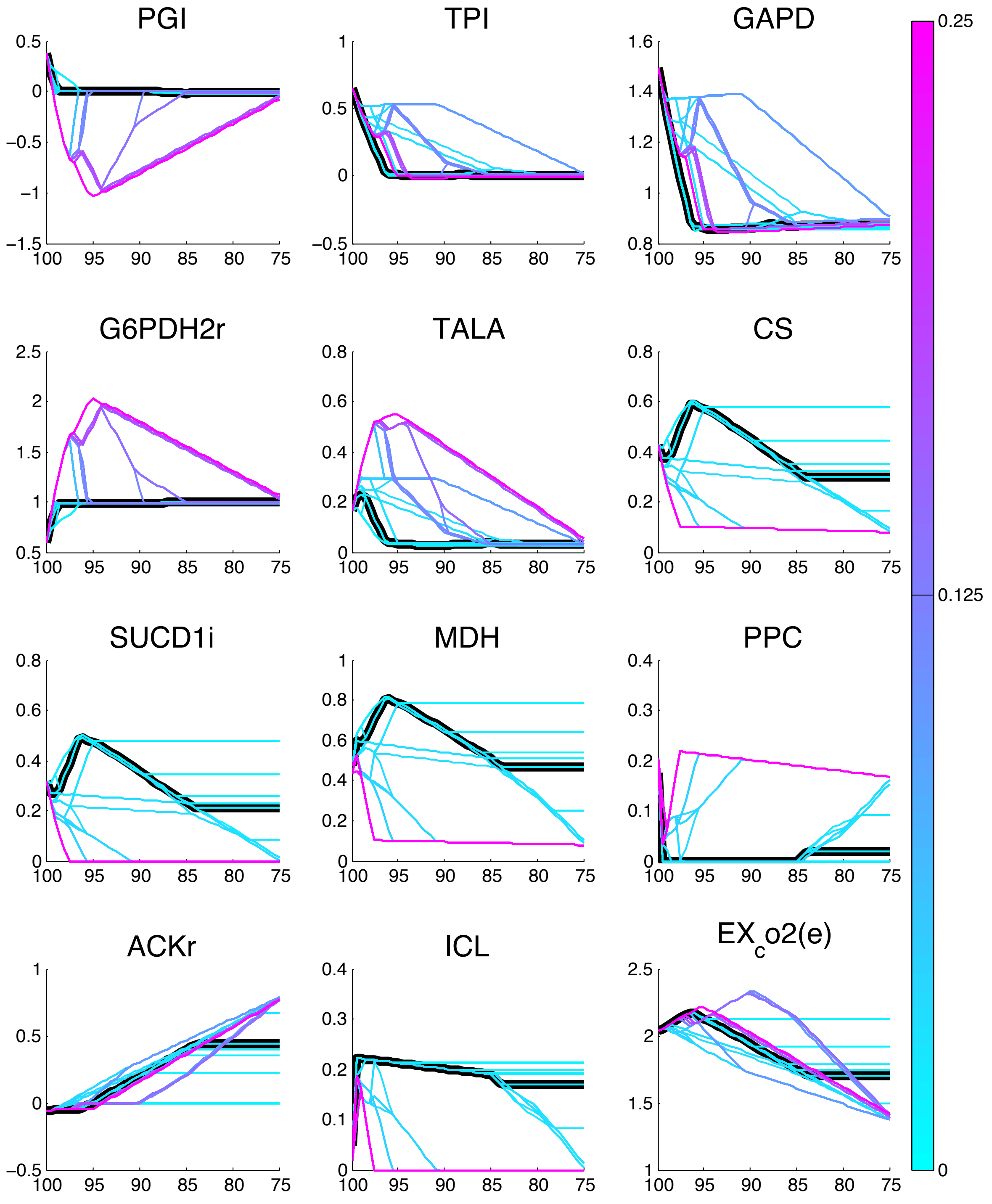

SI Figure 1 A: Model simulations for 251 values of α ranging from 0 to 0.25 $\mathrm{RT}\,\frac{\mathrm{mol}}{\mathrm{kcal}}$ in 10^{-2} increments using thermodynamic costs only. Higher values of α yield extremely high costs and give errors during the simulations. Varying values of α are plotted according to the color scale, and the line plotted in black indicates the chosen value of 0.02 $\mathrm{RT}\,\frac{\mathrm{mol}}{\mathrm{kcal}}$. We can see simulations are arranged in groups, given that many of the 251 simulations overlap. For high values of α the model converges to a flux through the pentose phosphate pathway and PPC reaction, while no flux through the TCA cycle or glyoxylate shunt are observed.

Combined Costs

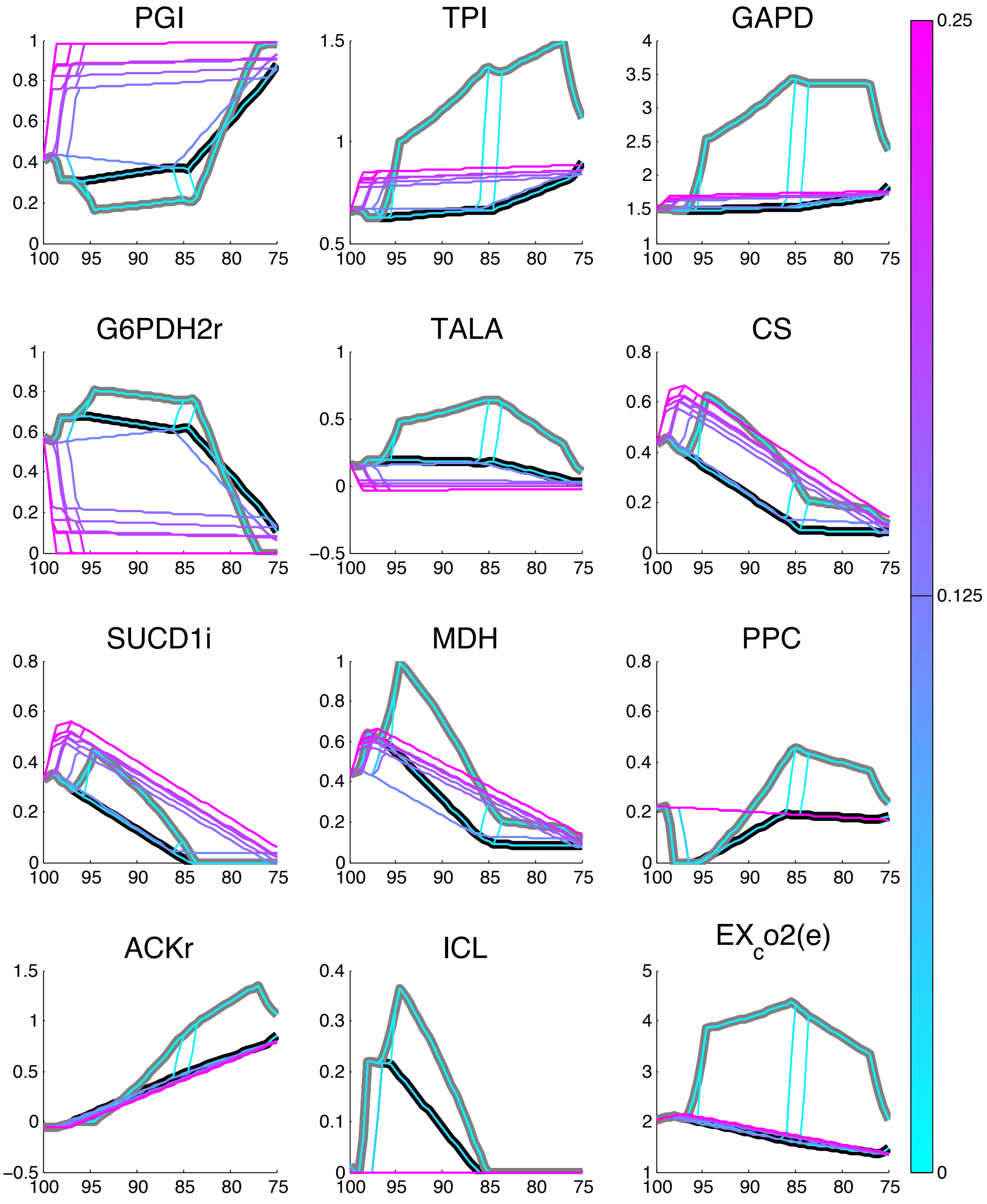

SI Figure 1 B: Model simulations for 251 values of α ranging from 0 to 0.25 $\mathrm{RT}\,\frac{\mathrm{mol}}{\mathrm{kcal}}$ in 10^{-2} increments using the combined cost function. Varying values of α are plotted according to the color scale, the line plotted in black indicates the chosen value of 0.02 $\mathrm{RT}\,\frac{\mathrm{mol}}{\mathrm{kcal}}$ and the line plotted in gray indicates the value of α=0 (or molecular weights cost only). We can see simulations are again arranged in groups, given that many of the 251 simulations overlap.
The value of α=0.02 was chosen in order to combine the molecular weight and thermodynamic costs, yielding intermediate fluxes through the pentose phosphate pathway while maintained the reported fluxes through PPC and ICL. While fluxes through the TCA cycle, particularly in the reactions CS and SUCD1i, are lower at α=0.02, all simulated values through that pathway are considerably lower than the reported data values.
