# Supplementary material for: Predicting internal cell fluxes at sub-optimal growth
Source: BMC Syst Biol. 2015 Apr 3;9:18. doi: 10.1186/s12918-015-0153-3 (PMC4397736; doi:10.1186/s12918-015-0153-3)
Supplement: Additional file 2 — Gibbs free energy change distribution. File showing plot and distribution of the Gibbs free energy change of reactions considered. [file 12918_2015_153_MOESM2_ESM.pdf]

Appendix 1

Calculation of Thermodynamic Costs

For visualization purposes, we will plot values on the *E. coli* reconstruction iJR904[1] Central Carbon Metabolism map downloaded from the BiGG database[2]. **Appendix 1 Figure 1** shows this maps and some of their general features for future reference. For more details such as specific reaction and metabolites please refer to the BiGG database website (bigg.ucsd.edu).

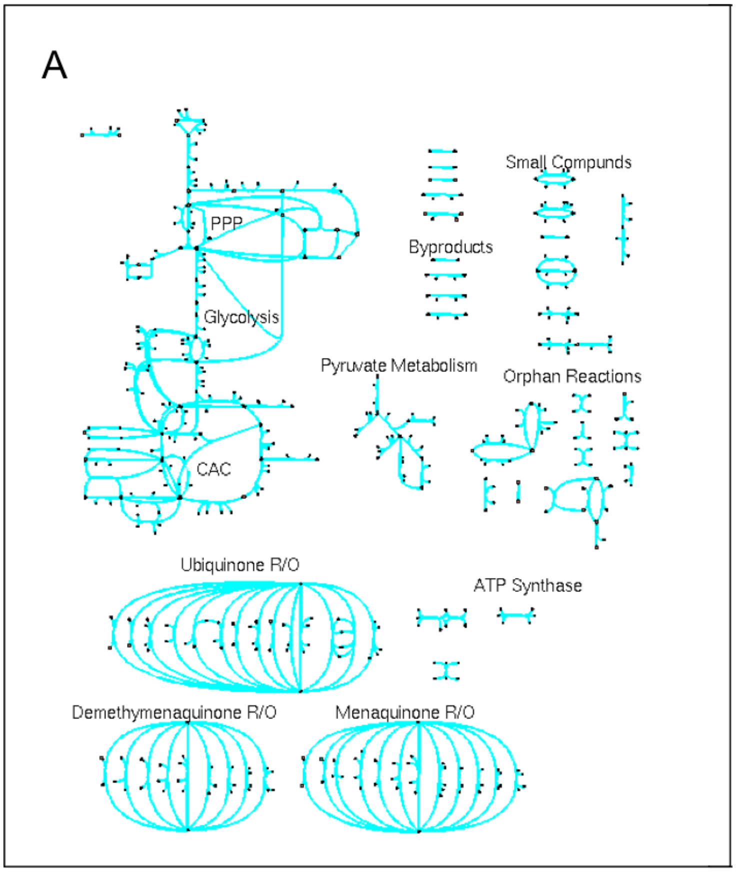

Appendix 1 Figure 1: Reference map for *E. coli* central metabolism based on the iJR904 reconstruction. Abbreviations are Citric acid cycle (CAC), Pentose phosphate pathway (PPP) and Reduction/Oxidation (R/O). For map details please refer to the BiGG database website (bigg.ucsd.edu).

In order to estimate the standard Gibbs free energy change of reactions in the model, the Gibbs energy of formation of 592 of the 625 model metabolites (94.72%) were extracted from the MetaCyc Database[3]. These values were in turn calculated using a group contribution technique[4] and adapted for an ionic strength of 0.25 and a pH of 7.3[5]. Using these values, the Gibbs free energy change of each reaction was estimated as the difference in energy of formation between products and reactants. Gibbs free energy change of reactions involving the metabolites for which Gibbs energy of formation were not found were set to zero. The values used for each metabolite as well as the resulting free energy change for reversible reactions can be seen in **SI table 1**. Values are expressed in kcal/mol. **Appendix 1 Figure 2** plots onto

the reference map the standard Gibbs free energy change of reversible reactions in the central carbon metabolism.

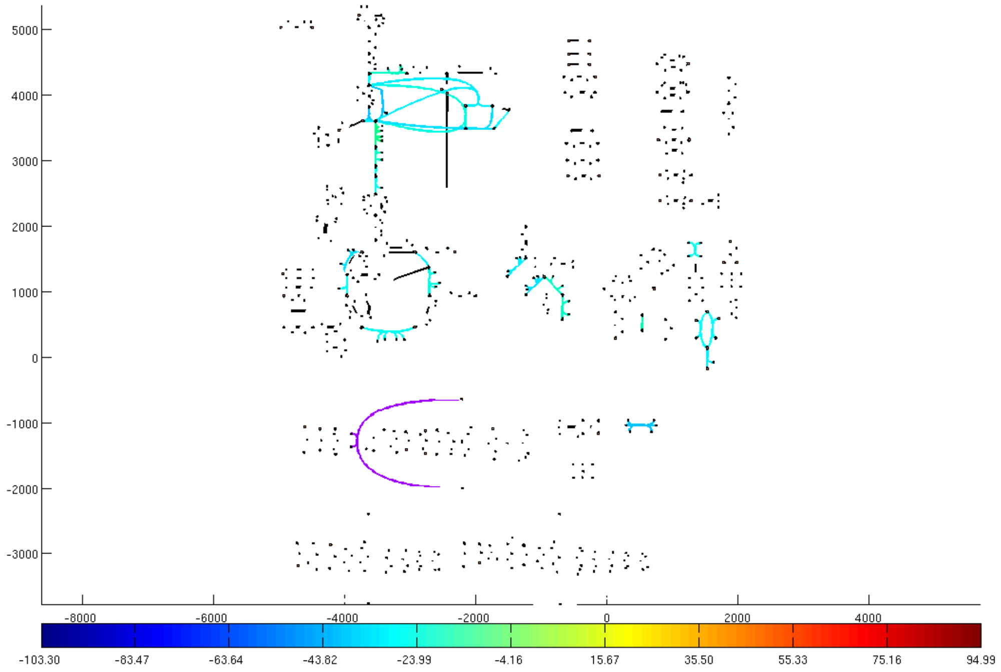

Appendix 1 Figure 2: Standard Gibbs free energy change for reversible reaction in the Central Carbon Metabolism.

We have also plotted a histogram of the Standard Gibbs free energy change of all the reversible reactions in the model (**appendix 1 figure 3**).

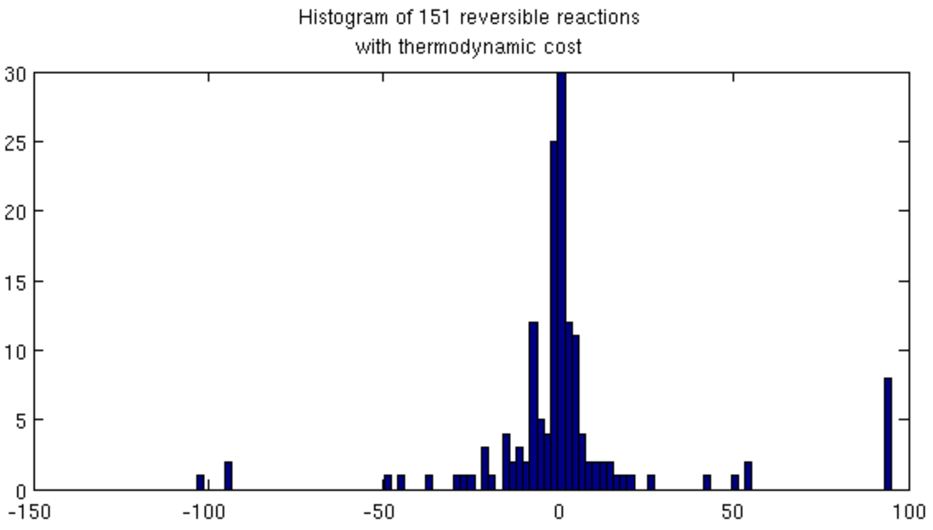

Appendix 1 Figure 3: Standard Gibbs free energy change distribution of all reversible model reactions.

References

1. Reed J.L., Vo T.D., Schilling C.H. and Palsson B.Ø. **An expanded genome-scale model of Escherichia coli K-12 (iJR904 GSM/GPR)**. *Genome Biol*, 2003. 4:R54.
2. Schellenberger, J., Park, J.O., Conrad, T.C., and Palsson, B.Ø., **BiGG: a Biochemical Genetic and Genomic knowledgebase of large scale metabolic reconstructions**. *BMC Bioinf*, 2010. **11**:213.
3. Caspi T. et. al. **The MetaCyc database of metabolic pathways and enzymes and the BioCyc collection of Pathways/Genome Databases**. *Nucl Acids Res*, 2014. 42:D459-471
4. Janikowski M.D., Henry, C.S., Broadbelt L.J. and Hatzimanikatis V. **Group contribution method for thermodynamics analysis of complex metabolic networks**. *Biophys J.* 2008. 95(3) 1487-99.
5. Alberty R.A. **Thermodynamics of Biochemical Reactions**. *Wiley InterScience*. 2003.
