# Supplementary material for: Predicting internal cell fluxes at sub-optimal growth
Source: BMC Syst Biol. 2015 Apr 3;9:18. doi: 10.1186/s12918-015-0153-3 (PMC4397736; doi:10.1186/s12918-015-0153-3)
Supplement: Additional file 3 — Molecular weight and cost calculation. Details on how Molecular weight values were obtained and final costs calculated. Molecular weights and final costs distribution are also plotted. [file 12918_2015_153_MOESM3_ESM.pdf]

Appendix 2

Enzyme size calculation and cost distribution.

For visualization purposes, we will plot values on the *E. coli* reconstruction iJR904[1] Central Carbon Metabolism map downloaded from the BiGG database[2]. **Appendix 2 Figure 1** shows this maps and some of their general features for future reference. For more details such as specific reaction and metabolites please refer to the BiGG database website (bigg.ucsd.edu).

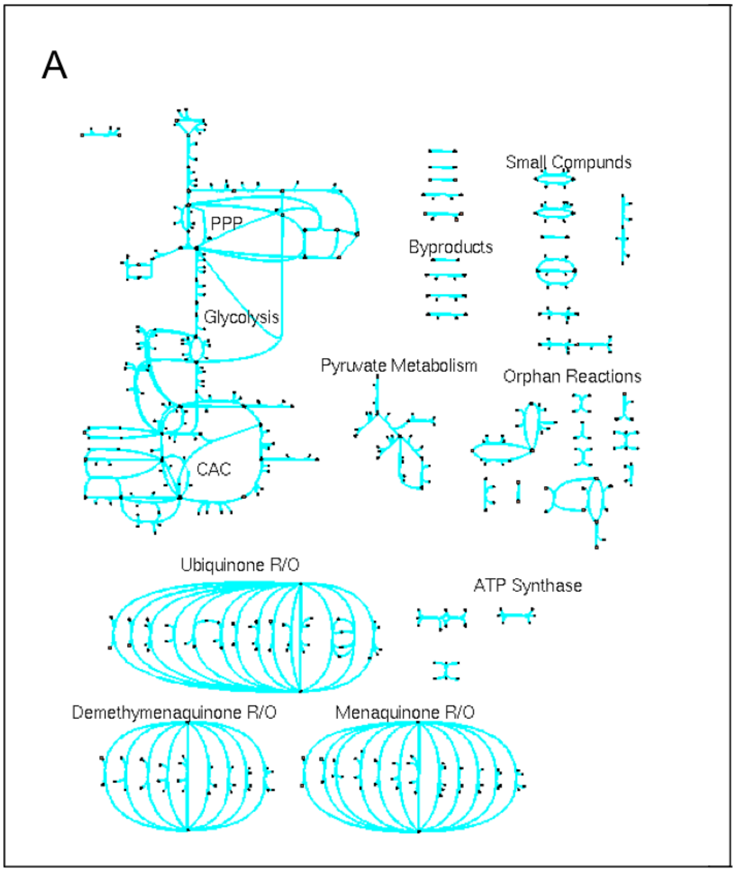

Appendix 2 Figure 1: Reference map for *E. coli* central metabolism based on the iJR904 reconstruction. Abbreviations are Citric acid cycle (CAC), Pentose phosphate pathway (PPP) and Reduction/Oxidation (R/O). For map details please refer to the BiGG database website (bigg.ucsd.edu).

Molecular weight values were estimated 506 of the 523 enzyme associated model reactions. All but three of these values were extracted directly from the BRENDA database[3]. Values for *E. coli* were used whenever available. If such values were not available the value for the organism taxonomically closest to *E. coli* was used. The remaining three values were extracted from the EcoCyc[4] database. Five molecular weight values were added to the model where no associate E.C. number was included in the reconstruction. These five enzymes were:

- **PDH** (Pyruvate Dehydrogenase) - According to EcoCyc[4] the complex has the composition $[(AceE)_2]_{12}[AceF]_{24}[(Lpd)_2]_6$. These subunits have molecular weights of 99.7 (AceE), 66.1 (AceF) and 56 (Lpd). Considering the AceE dimer as the active site, we estimate this complex to have 12 active sites. The molecular weight by active site was then estimated to be ((24*99.7)+(24*66.1)+(12*56))/12 = 387.6 kDa.
- **AKGDH** (α-Ketoglutarate Dehydrogenase Complex) - According to two studies the molecular weight of this complex is 2470 kDa. This value is supported by EcoCyc which estimates the composition of the complex to be $[(SucA)_{12}][(SucB)_{24}][(Lpd)_2]$. With subunits molecular weights of 105.6 (SucA), 44 (SucB) and 56 (Lpd), the weight of the complex is estimated to be (105.6*12)+(44*24)+(56*2) = 2435.2 kDa. Considering the complex to have 12 active sites (SucA), the molecular weight per active site was considered to be 2470/12 = 205.83 kDa.
- **F6PA** had no associated E.C number in the model downloaded. According to Metacyc[5], fructose 6-phosphate aldolase 1 is composed of 10 *FsaA* subunits, each with a weight of 24.0 kDa. This enzyme weight was then added as 240kDa.
- **CS** citrate synthase also had no associated EC number. We have chosen to include the enzyme 2.3.3.16, which is the Citrate Synthase with unknown stereospecificity. This enzyme contains a lot more information, and is the only form of Citrate Synthase with a know *E. coli* molecular weight. The value of 269 kDa was extracted from BRENDA.
- **PFL** also contained no associated EC number. This enzyme was looked up on BRENDA and we found it to be 2.3.1.54. Its molecular weight was calculated to be the median between six reported values for *E. coli* and was added to the model.

All values used in this study are reported in **SI table 1**. For the remaining 17 enzyme associated reactions, where molecular weight values were not found, molecular weights equal to the median of the calculated values were given.

Appendix Figure 2 presents the distribution of the organisms from where the molecular weights were extracted, according to the *E. coli* taxonomy tree. For each level specified in **appendix 2 figure 2** we have the number of molecular weights that share that as the highest level of similarity to *E. coli*. For example, the molecular weights of 15 proteins were extracted from organisms that are also proteobacteria but are not gammaproteobacteria. The complexes Pyruvate Dehydrogenase and α-Ketoglutarate Dehydrogenase, as well as all the values extracted from EcoCyc, are accounted for under *E. coli*.

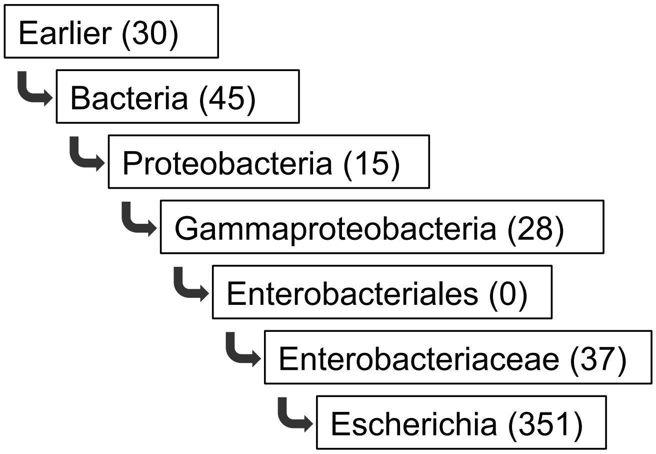

Appendix 2 Figure 2: Taxonomy tree of *E. coli*. Numbers represent the number of molecular weight values that were extracted from organisms that share that as the highest level of similarity to *E. coli*.

For visualization purposes we also plotted the molecular weights on the reference map (**appendix 2 figure 3**), as well as a histogram of all the molecular weight values used (**appendix 2 figure 4**).

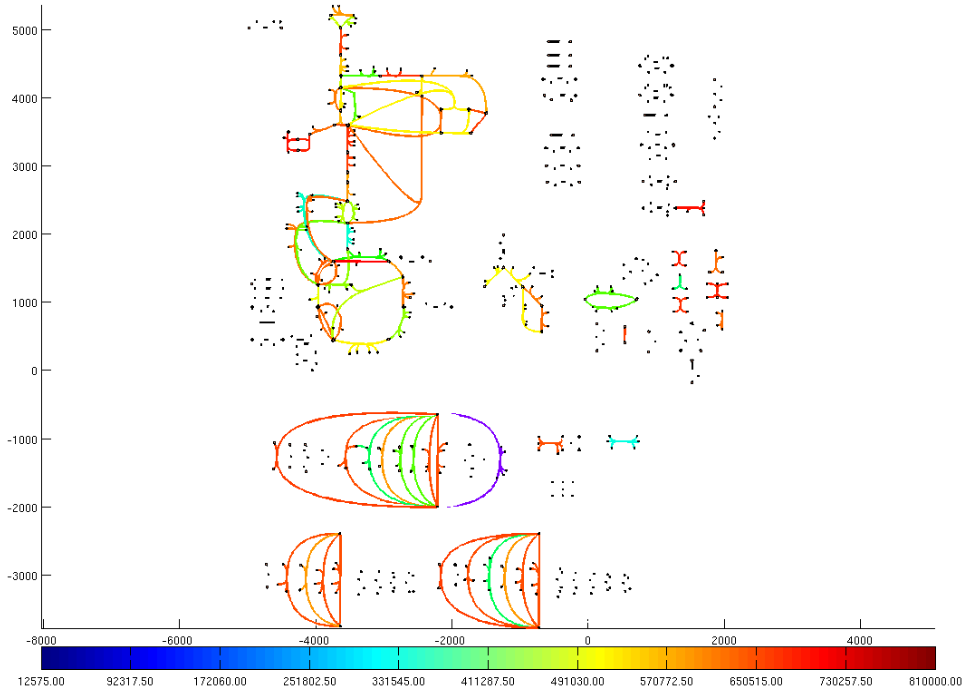

Appendix 2 Figure 3: Molecular weights used for all enzymes in the central carbon metabolism. Reactions in white are reactions for which no enzyme was associated.

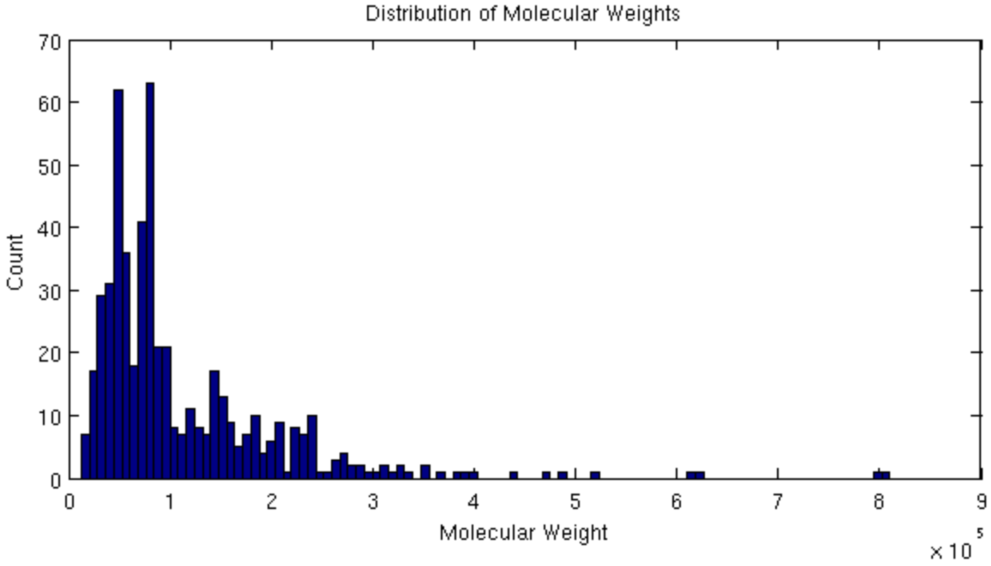

Appendix 2 Figure 4: Histogram of all molecular weight values used in the cost distribution.

Using the Gibbs free energy change values described in **Appendix 1** and presented in **SI table** 1, we calculate the thermodynamic cost for reversible reactions as described in the main text. The final combined costs are displayed here in the reference map (**appendix 2 figure 5**) and as a histogram (**appendix 2 figure 6**). Costs for the reversed reaction are also shown separately. Such costs stem from the calculation of the cost using the opposite Gibbs energy change value, and are shown only for reversible reactions (reactions fo which this cost was applied).

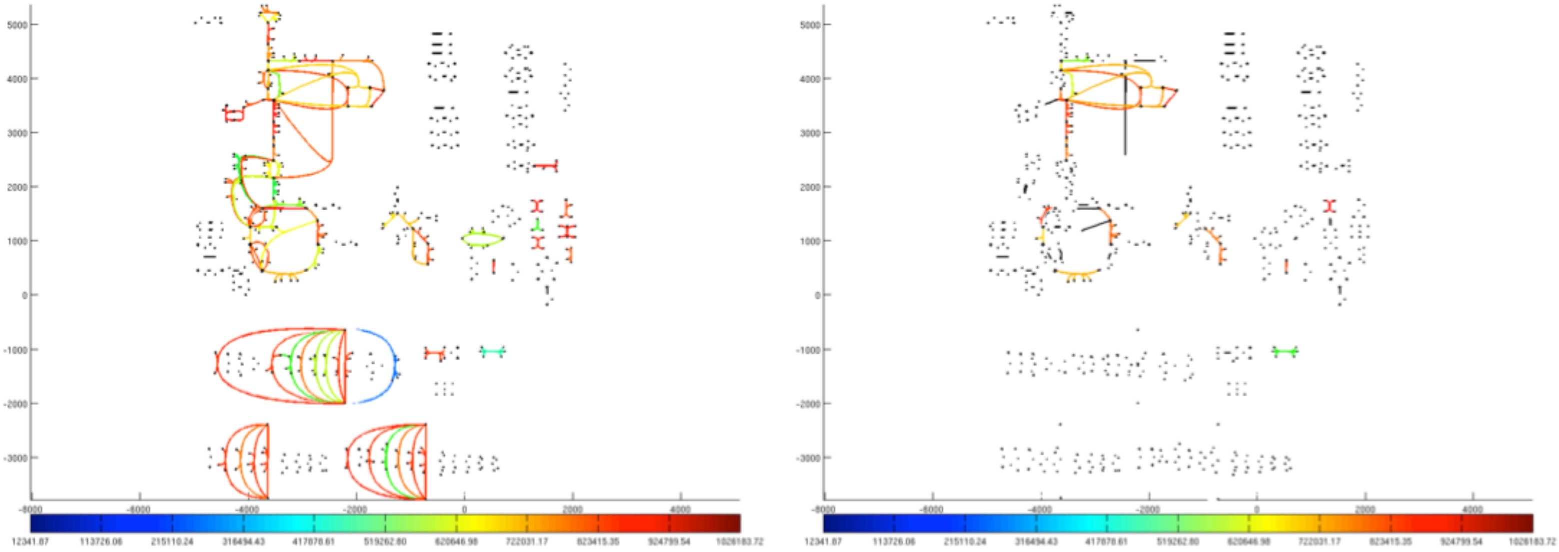

Appendix 2 Figure 5: Final cost used, combining molecular weights and thermodynamic penalty, for all reactions in the central carbon metabolism. Map in the left shows costs used in the forward direction, while map on the right shows the cost used in the backwards reaction for reversible reactions.

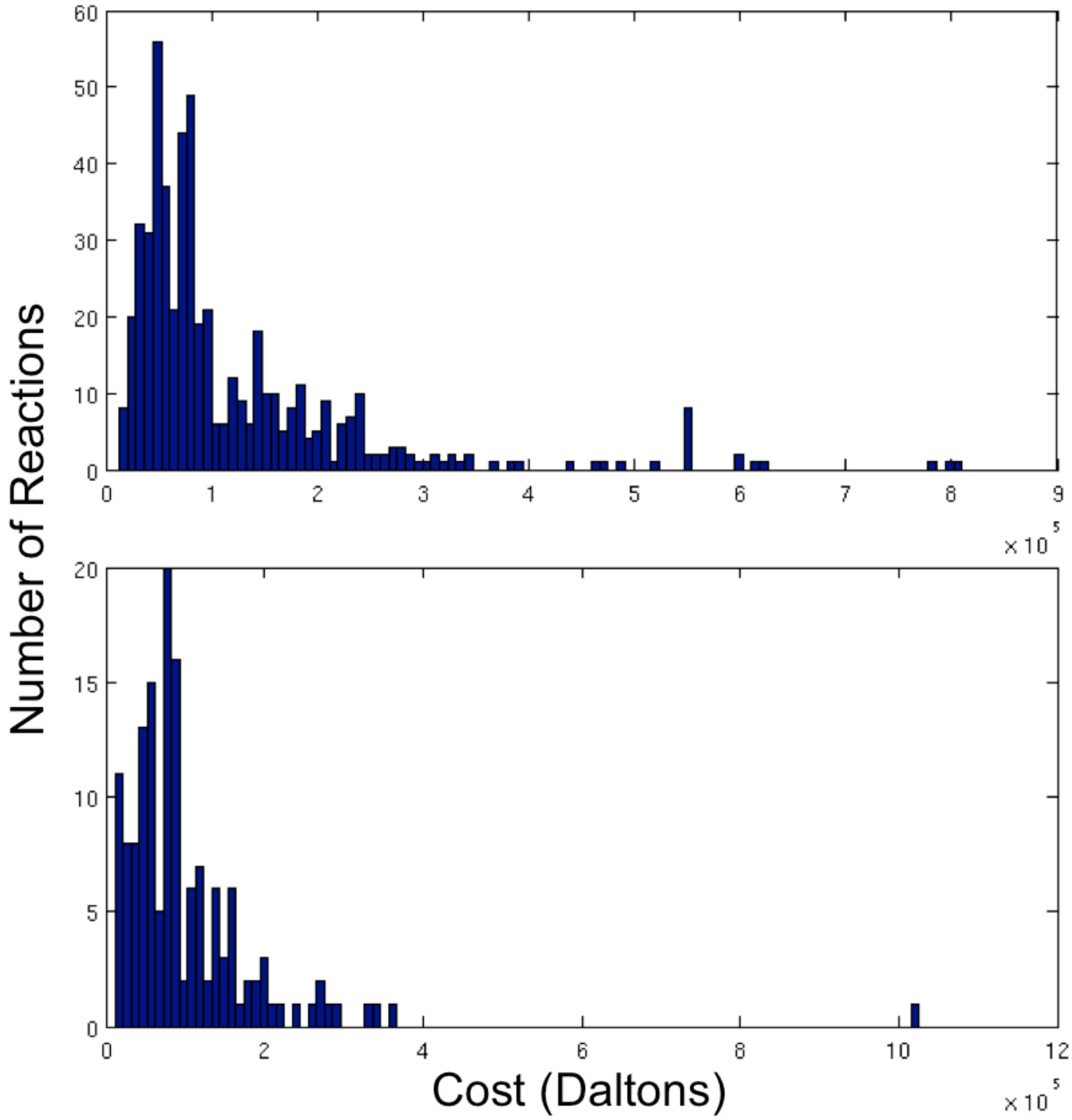

Appendix 2 Figure 6: Histogram of all costs used in the model for all forward (top) and backward (bottom) reaction rates. Backward rates are shown for the reverse rate of reversible reactions only.

References:

1. Reed J.L., Vo T.D., Schilling C.H. and Palsson B.Ø. **An expanded genome-scale model of Escherichia coli K-12 (iJR904 GSM/GPR)**. *Genome Biol*, 2003. 4:R54.
2. Schellenberger, J., Park, J.O., Conrad, T.C., and Palsson, B.Ø., **BiGG: a Biochemical Genetic and Genomic knowledgebase of large scale metabolic reconstructions**. *BMC Bioinf*, 2010. **11**:213.
3. Schomburg, I., Chang, A. and Schomburd, D. **Standardization in enzymology-Data integration in the world's enzyme information system BRENDA**. Perspectives in Science. 2014. (1)15-23.
4. Keseler et al. **EcoCyc: fusing model organism databases with systems biology**. Nucleic Acid Research, 2103. 41: D605-12.
5. Caspi T. et. al. **The MetaCyc database of metabolic pathways and enzymes and the BioCyc collection of Pathways/Genome Databases**. *Nucl Acids Res*, 2014. 42:D459-471
