# Supplementary material for: Predicting internal cell fluxes at sub-optimal growth
Source: BMC Syst Biol. 2015 Apr 3;9:18. doi: 10.1186/s12918-015-0153-3 (PMC4397736; doi:10.1186/s12918-015-0153-3)
Supplement: Additional file 5 — Fundamental pathway analysis. Example demonstrating the recovery of Extreme Pathways by adding reactions loops back into the Fundamental Pathways. Details on how the fundamental pathway analysis presented in the manuscript was performed are also available. [file 12918_2015_153_MOESM5_ESM.pdf]

Appendix 3

Pathway Recovery by Reintroduction of Loops

For this analysis we calculate both standard *Extreme Pathways* and *Fundamental Pathways* defined after deleting currency metabolites from the model. We then identified a series of loops and reactions in the original model which can be used to counteract the imbalance caused by the *Fundamental Pathways*. These loops and reactions were then added to the *Fundamental Pathways* in order to recover the original *Extreme Pathways*. The model tailoring and recovery process are described below.

Model Tailoring

For this analysis we used the *E. coli* textbook model[1] with different sets of inputs and outputs. The metabolites deleted from the model are:

- ADP (**adp[c]**);
- AMP (**amp[c]**);
- ATP (**atp[c]**);
- CO2 (**co2[c]**); CO2 (**co2[e]**);
- H2O (**h2o[c]**); H2O (**h2o[e]**);
- H+ (**h[c]**); H+ (**h[e]**);
- Nicotinamide adenine dinucleotide (**nad[c]**);
- Nicotinamide adenine dinucleotide - reduced (**nadh[c]**);
- Nicotinamide adenine dinucleotide phosphate (**nadp[c]**);
- Nicotinamide adenine dinucleotide phosphate - reduced (**nadph[c]**);
- O2 (**o2[c]**); O2 (**o2[e]**);
- Phosphate (**pi[c]**); Phosphate (**pi[e]**);
- Ubiquinone-8 (**q8[c]**);
- Ubiquinol-8 (**q8h2[c]**).

With that, the reactions deleted from the model are:

- **ADK1:** amp[c] + atp[c] <=> 2 adp[c]
- **ATPM:** atp[c] + h2o[c] <=> adp[c] + h[c] + pi[c]
- **ATPS4r:** adp[c] + 4.000000 h[e] + pi[c] <=> atp[c] + h2o[c] + 3 h[c]
- **Biomass_Ecoli_core_N(w/GAM)_Nmet2:** 1.496000 3pg[c] + 3.747800 accoa[c] + 59.810000 atp[c] + 0.361000 e4p[c] + 0.070900 f6p[c] + 0.129000 g3p[c] + 0.205000 g6p[c] + 0.255700 gln-L[c] + 4.941400 glu-L[c] + 59.810000 h2o[c] + 3.547000 nad[c] + 13.027900 nadph[c] + 1.786700 oaa[c] + 0.519100 pep[c] + 2.832800 pyr[c] + 0.897700 r5p[c] -> 59.810000 adp[c] + 4.118200 akg[c] + 3.747800 coa[c] + 59.810000 h[c] + 3.547000 nadh[c] + 13.027900 nadp[c] + 59.810000 pi[c]
- **CO2t:** co2[e] <=> co2[c]
- **CYTBD:** 2 h[c] + 0.5 o2[c] + q8h2[c] -> h2o[c] + 2 h[e] + q8[c]
- **EX_co2(e):** co2[e] <=>
- **EX_h2o(e):** h2o[e] <=>
- **EX_h(e):** h[e] <=>
- **EX_o2(e):** o2[e] <=>

- **EX_pi(e):** pi[e] <=>
- **H2Ot:** h2o[e] <=> h2o[c]
- **NADH16:** 4 h[c] + nadh[c] + q8[c] -> 3 h[e] + nad[c] + q8h2[c]
- **NADTRHD:** nad[c] + nadph[c] -> nadh[c] + nadp[c]
- **O2t:** o2[e] <=> o2[c]
- **PIt2r:** h[e] + pi[e] <=> h[c] + pi[c]
- **THD2:** 2 h[e] + nadh[c] + nadp[c] -> 2 h[c] + nad[c] + nadph[c]

We have verified that the considerations presented in the manuscript are satisfied in this reconstruction. That is, no reaction aside from the exchange reactions are sinks or sources, all metabolites deleted are completely removed from all reactions and compartments of the model, and no reaction deleted contains a metabolite not deleted from the model.

Loops Definition

Extreme Pathways were then calculated for the original model and *Fundamental Pathways* were calculated for the tailored model. Several loops were then added to the Fundamental Pathways in order to account for the imbalance those reactions cause in the original model. We find that most *Extreme Pathways* can be recovered by the addition of the loops we found.

First, we found the model has only one way to balance some of the currency metabolites removed (All reactions mentioned below can be found in the list of deleted reactions or in the *E. coli textbook* model[1]):

- AMP imbalances are balanced by the ADK1 reaction (amp[c] + atp[c] <=> 2 adp[c]) and converted into ATP/ADP imbalances.
- Q8H2/Q8 imbalances are balanced by the CYTBD reaction (2 h[c] + 0.5 o2[c] + q8h2[c] -> h2o[c] + 2 h[e] + q8[c]), and converted into a proton gradient and water imbalance.
- NADP+/NADPH imbalances are balanced by the reactions THD2 (2 h[e] + nadh[c] + nadp[c] -> 2 h[c] + nad[c] + nadph[c]) or NADTRHD (nad[c] + nadph[c] -> nadh[c] + nadp[c]) and converted into an NAD+/NADH imbalance and possibly a proton gradient (in the case of THD2).
- NAD+/NADH imbalances are balanced by the NADH6 (4.5 h[c] + nadh[c] + q8[c] -> 3.5 h[e] + nad[c] + q8h2[c]) reaction and converted into a Q8H2/Q8 and proton gradient imbalance. the Q8H2/Q8 imbalance is then balanced by the CYTBD reaction.

Some imbalances are then accounted for by these reactions. Others such as O_2, H_2O and CO_2 can be counterbalanced by the exchange reactions and membrane transport reactions directly. We then find that only two imbalances can be balanced by multiple model loops: ATP hydrolysis and transport of protons across the cell membrane.

In order to recover the *Extreme Pathways* from the *Fundamental Pathways*, we initially added the reactions mentioned above with the appropriate fluxes in order to balance all costs except for ATP hydrolysis and transfer of protons across the cell membrane. We then sequentially added several combinations of loops and reactions back into the model in order to balance the remaining currency metabolites. We performed several cycles of loop addition back into the model, where in each cycle all combinations ATP hydrolysis and proton gradient balancing were attempted. We then compared the *Extreme Pathways* to see if any of them were equal to the pathways obtained by adding loops back into the model. Each cycle of loop addition is described in the table below:

Loop Addition Cycle	ATP balancing loops	H gradient balancing loops
1	• ATPS4r	• NADTRHD and THD2<br>• SUCCt2_2 and SUCCt3<br>• FORt2 and FORti<br>• ATPS4r and ATPM
2	• ATPM	• NADTRHD and THD2<br>• SUCCt2_2 and SUCCt3<br>• FORt2 and FORti<br>• ATPS4r and ATPM
3	• GLNS and GLUN<br>• FBP and PFK<br>• GLNS, GLUDy and GLUSy	• NADTRHD and THD2<br>• SUCCt2_2 and SUCCt3<br>• FORt2 and FORti<br>• ATPS4r and ATPM
4	• PPC and PPCK<br>• ADK1, PPS and PYK	• NADTRHD and THD2<br>• SUCCt2_2 and SUCCt3<br>• FORt2 and FORti<br>• ATPS4r and ATPM
5	• ATPM	• ATPS4r, GLNS, GLUDy and GLUSy<br>• ATPS4r, FBP and PFK<br>• ATPS4r, PPC and PPCK<br>• ADK1, ATPS4r, PPS and PYK<br>• ATPS4r, GLNS and GLUN
6	• GLNS and GLUN<br>• FBP and PFK<br>• GLNS, GLUDy and GLUSy	• ATPS4r, GLNS, GLUDy and GLUSy<br>• ATPS4r, FBP and PFK<br>• ATPS4r, PPC and PPCK<br>• ADK1, ATPS4r, PPS and PYK<br>• ATPS4r, GLNS and GLUN
7	• PPC and PPCK<br>• ADK1, PPS and PYK	• ATPS4r, GLNS, GLUDy and GLUSy<br>• ATPS4r, FBP and PFK<br>• ATPS4r, PPC and PPCK<br>• ADK1, ATPS4r, PPS and PYK<br>• ATPS4r, GLNS and GLUN
8	• ADK1, MDH, ME1, PPC and PPS<br>• ADK1, MDH, ME2, NADTRHD, PPC and PPS<br>• MDH, ME2, NADTRHD, PPC and PYK<br>• MDH, ME1, PPC and PYK	• NADTRHD and THD2<br>• SUCCt2_2 and SUCCt3<br>• FORt2 and FORti<br>• ATPS4r and ATPM<br>ATPM
9	• ADK1, PPS and PYK<br>• PPC and PPCK<br>• GLNS and GLUN<br>• FBP and PFK<br>• ADK1, MDH, ME1, PPC and PPS<br>• GLNS, GLUDy and GLUSy<br>• ATPS4r<br>• ADK1<br>• MDH, ME2, NADTRHD, PPC and PPS<br>• ATPM<br>• MDH, ME2, NADTRHD, PPC and PYK<br>• MDH, ME1, PPC and PYK	• ADK1, ATPS4r, MDH, ME1, PPC and PPS<br>• ADK1, ATPS4r, MDH, ME2, NADTRHD, PPC and PPS

Appendix 3 Table 1: Loops added back into the model in each cycle of Pathway recovery. ATP balancing loops were added back with the appropriate fluxes in order to cancel any ATP, ADP and Pi imbalance, then the proton gradient loops were added in order to transport protons across the cell membrane. For each cycle of loop addition all combinations of ATP and proton gradient balancing were attempted.

After these loops were added we also balanced ATP imbalances from one fundamental pathway using another fundamental pathway. That is, if one pathway consumes ATP and another produces it, we combined these in a ratio such that the ATP imbalance was cancelled out. Any remaining proton gradient imbalance was then balanced using the loops above.

Extreme Pathway and *Fundamental Pathway* Analysis were performed for two sets of exchange reactions, and the pathway recovery analysis described above was performed.

One input and one output.

We first allowed the model to uptake glucose and secrete ethanol only. With these sets of reactions we calculated 1188 *Extreme Pathways*, and after tailoring the model as we have described above, 59 *Fundamental Pathways*. We removed from the model 11 *Extreme Pathways* that did not exchange glucose or ethanol (*Type III Pathways*). By adding back the reaction loops described above we were able to recover 1147 (97.45%) of the original *Extreme Pathways*. The distributions are as described in the following table and figure. All Pathways and pathway costs can be found in the **SI Table 2**.

Loop Addition cycle	Number of Pathways Recovered
1	196
2	138
3	351
4	162
5	14
6	138
7	68
8	48
9	20
combination	12

Appendix 3 Table 2: Number of pathways recovered during each cycle of loop addition for a model with one input (glucose) and one output (ethanol).

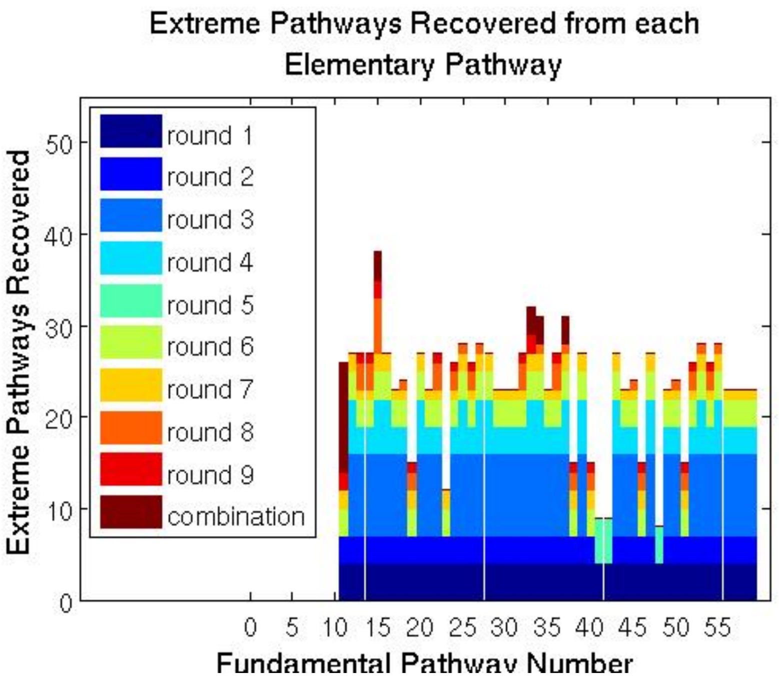

Appendix 3 Figure 1: Distribution of pathways recovered in each cycle of loop addition for model with one input (glucose) and one output (ethanol). X-axis represents each *Fundamental Pathway*, and Y-axis indicates how many *Extreme Pathways* were recovered by using the given *Fundamental Pathway* as backbone reactions.

One input and two freely exchanged byproducts

Next we performed the same analysis for a different set of exchange reactions. This time the model was allowed to uptake glucose and exchange ethanol and lactate freely. We found 2222 *Extreme Pathways*, 11 of which did not exchange any of these metabolites (*Type III Pathways*). After tailoring the model we found 114 *Fundamental Pathways*. Using the same techniques described above we were able to recover 2157 (97.56%) of the original *Extreme Pathways*. Distribution of such recoveries are presented below, and again all Pathways and pathway costs can be found in the **SI Table 2**.

Loop Addition cycle	Number of Pathways Recovered
1	416
2	264
3	558
4	244
5	64
6	264
7	128
8	74
9	40
combination	105

Appendix 3 Table 3: Number of pathways recovered during each cycle of loop addition for a model with one input (glucose) and two freely exchanged byproducts (ethanol and lactate).

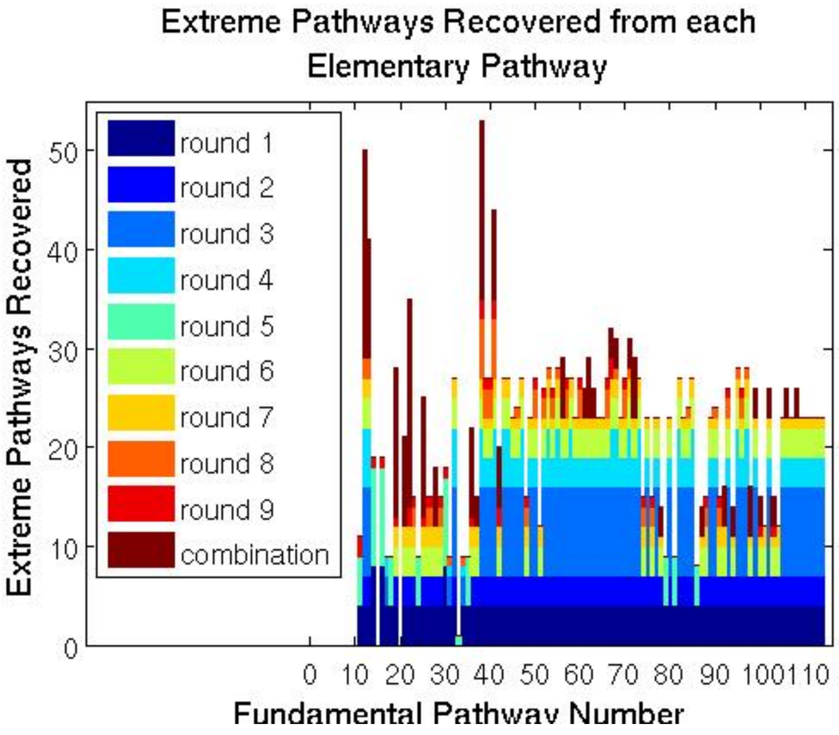

Appendix 3 Figure 2: Distribution of pathways recovered in each cycle of loop addition for model with one input (glucose) and two freely exchangeable byproducts (ethanol and lactate). X-axis represents each *Fundamental Pathway*, and Y-axis indicates how many pathways were recovered by using the given *Fundamental Pathway* as backbone reactions.

Conclusions

We can see that most *Extreme Pathways* can be recovered by adding the model redundancies back into the *Fundamental Pathways*. The pathways not recovered could possibly be reversed by adding different *Fundamental Pathways* in different ways, or adding back into the model more complex loops. The recovery of over 97% of *Extreme Pathways*, however, we believe is sufficient for a proof of principle.

Fundamental Pathway Analysis of *E. coli* Central Metabolism

Model Tailoring and Pathway Calculation

In our manuscript we calculated *Fundamental Pathways* for a system containing all reactions in the Central Carbon Metabolism of the *E. coli iJR904* reconstruction[2]. Reactions included in the model are all the reactions included in the Central Carbon Metabolism map downloaded from the BiGG database[3]. The exchange reactions *EX_ac(e)*, *EX_etoh(e)*, *EX_glc(e)* and *EX_lac_D(e)* as well as the transport reactions *GLCpts* and *GLCt2* were also included for inputs and outputs in the model. We defined glucose as an input and ethanol, lactate and acetate were allowed to be exchanged freely.

For the *Fundamental Pathways* analysis the following metabolites were deleted:

- 2-Demethylmenaquinone 8(**2dmmq8[c]**);
- 2-Demethylmenaquinol 8(**2dmmql8[c]**);
- ADP(adp[c]); AMP(amp[c]);
- ATP(**atp[c]**);
- CO2(**co2[c]**); CO2(**co2[e]**);
- Flavin adenine dinucleotide oxidized(**fad[c]**);
- Flavin adenine dinucleotide reduced(**fadh2[c]**);
- Fe2+(**fe2[c]**); Fe2+(**fe2[e]**);
- Formate(**for[c]**); Formate(**for[e]**);
- H2(**h2[c]**);
- Hydrogen peroxide(**h2o2[c]**);
- H2O(**h2o[c]**); H2O(**h2o[e]**);
- H+(**h[c]**); H+(**h[e]**);
- Bicarbonate(**hco3[c]**);
- potassium(**k[c]**); potassium(**k[e]**);
- Menaquinol 8(**mql8[c]**);
- Menaquinone 8(**mqn8[c]**);
- Sodium(**na1[c]**); Sodium(**na1[e]**);
- Nicotinamide adenine dinucleotide(**nad[c]**);
- Nicotinamide adenine dinucleotide - reduced(**nadh[c]**);
- Nicotinamide adenine dinucleotide phosphate(**nadp[c]**);
- Nicotinamide adenine dinucleotide phosphate - reduced(**nadph[c]**);
- Nitrite(**no2[c]**); Nitrite(**no2[e]**);
- Nitrate(**no3[c]**); Nitrate(**no3[e]**);
- O2(**o2[c]**); O2(**o2[e]**);
- Superoxide anion(**o2s[c]**);
- Phosphate(**pi[c]**); Phosphate(**pi[e]**);
- Diphosphate(**ppi[c]**);

- Ubiquinone-8(**q8[c]**);
- Ubiquinol-8(**q8h2[c]**);
- Sulfite(**so3[c]**).

The reactions that were deleted are:

- **ATPM:** atp[c] + h2o[c] -> adp[c] + h[c] + pi[c]
- **ATPS4r:** adp[c] + 4 h[e] + pi[c] <=> atp[c] + h2o[c] + 3 h[c]
- **CAT:** 2 h2o2[c] -> 2 h2o[c] + o2[c]
- **CO2t:** co2[e] <=> co2[c]
- **CYTBD:** 2 h[c] + 0.5 o2[c] + q8h2[c] -> h2o[c] + 2 h[e] + q8[c]
- **CYTBO3:** 2.5 h[c] + 0.5 o2[c] + q8h2[c] -> h2o[c] + 2.5 h[e] + q8[c]
- **FDH2:** for[c] + 3 h[c] + q8[c] -> co2[c] + 2 h[e] + q8h2[c]
- **FDH3:** for[c] + 3 h[c] + mqn8[c] -> co2[c] + 2 h[e] + mql8[c]
- **FE2abc:** atp[c] + fe2[e] + h2o[c] -> adp[c] + fe2[c] + h[c] + pi[c]
- **FHL:** for[c] + h[c] -> co2[c] + h2[c]
- **FORt:** for[e] <=> for[c]
- **H2Ot:** h2o[e] <=> h2o[c]
- **HCO3E:** co2[c] + h2o[c] <=> h[c] + hco3[c]
- **HYD1:** h2[c] + 2 h[c] + q8[c] -> 2.000000 h[e] + q8h2[c]
- **HYD2:** h2[c] + 2 h[c] + mqn8[c] -> 2 h[e] + mql8[c]
- **HYD3:** 2dmmq8[c] + h2[c] + 2 h[c] -> 2dmmql8[c] + 2 h[e]
- **Kabc:** atp[c] + h2o[c] + k[e] -> adp[c] + h[c] + k[c] + pi[c]
- **Kt2r:** h[e] + k[e] <=> h[c] + k[c]
- **NADH10:** h[c] + mqn8[c] + nadh[c] -> mql8[c] + nad[c]
- **NADH5:** h[c] + nadh[c] + q8[c] -> nad[c] + q8h2[c]
- **NADH6:** 4.5 h[c] + nadh[c] + q8[c] -> 3.5 h[e] + nad[c] + q8h2[c]
- **NADH7:** 3 h[c] + mqn8[c] + nadh[c] -> 2 h[e] + mql8[c] + nad[c]
- **NADH8:** 2dmmq8[c] + 3.8 h[c] + nadh[c] -> 2dmmql8[c] + 2.8 h[e] + nad[c]
- **NADH9:** 2dmmq8[c] + h[c] + nadh[c] -> 2dmmql8[c] + nad[c]
- **NADTRHD:** nad[c] + nadph[c] -> nadh[c] + nadp[c]
- **NAt3_1:** h[e] + na1[c] <=> h[c] + na1[e]
- **NAt3_15:** 3 h[e] + 2 na1[c] -> 3 h[c] + 2 na1[e]
- **NAt3_2 2:** h[e] + na1[c] -> 2 h[c] + na1[e]
- **NO2t2r:** h[e] + no2[e] <=> h[c] + no2[c]
- **NO3R1:** 2 h[c] + no3[c] + q8h2[c] -> h2o[c] + 2 h[e] + no2[c] + q8[c]
- **NO3R2:** 2 h[c] + mql8[c] + no3[c] -> h2o[c] + 2 h[e] + mqn8[c] + no2[c]
- **NO3t7:** no2[c] + no3[e] -> no2[e] + no3[c]
- **NTRIR2x:** 5 h[c] + 3 nadh[c] + no2[c] -> 2 h2o[c] + 3 nad[c] + nh4[c]
- **O2t:** o2[e] <=> o2[c]
- **PIabc:** atp[c] + h2o[c] + pi[e] -> adp[c] + h[c] + 2 pi[c]
- **PIt2r:** h[e] + pi[e] <=> h[c] + pi[c]
- **PPA:** h2o[c] + ppi[c] -> h[c] + 2 pi[c]
- **SPODM:** 2 h[c] + 2 o2s[c] -> h2o2[c] + o2[c]
- **SUCD4:** fadh2[c] + q8[c] <=> fad[c] + q8h2[c]
- **THD2:** 2 h[e] + nadh[c] + nadp[c] -> 2 h[c] + nad[c] + nadph[c]

The only consideration specified in the manuscript that was not met in this analysis is the fact that the reaction NTRIR2x was deleted and contains the metabolite nh4[c] (ammonium). We only allowed this model to exchange glucose, ethanol lactate and acetate, however, and no source of Nitrogen was introduced. With that, no nitrogen exchange was analyzed and therefore no pathway would have been excluded by removing this reaction.

A total of 4435 *Fundamental Pathways* were then calculated. 4418 of these pathways uptake glucose. These 4418 pathways and their relative costs can be found in the supplemental table **SI table 2**.

Path Energy and Cost Calculation

In order to estimate the energy potential of each pathway, we started with the ATP imbalance associated with each pathway. The ATP production potential of seven more currency metabolites was then calculated based on the lowest possible cost to transform each metabolite into ATP. The pathways used are described in **Appendix 3 Table 4**.

	**q8h2[c]**	**2dmmql8[c]**	**mql8[c]**	**fadh2[c]**	**for[c]**	**nadph[c]**	**nadh[c]**	**COST (kDA)**
**ATPS4r**	0.625	0.625	0.625	0.625	1.375	1.75	1.75	466
**CYTBO3**	1	1	1	1	1	1	1	0
**H2Ot**	1	1	1	1	1	1	1	0
**O2t**	0.5	0.5	0.5	0.5	0.5	0.5	0.5	0
**FRD3**	0	1	0	0	0	0	0	70
**SUCD1i**	0	1	1	0	0	0	0	70
**SUCD4**	0	1	1	1	0	0	0	0
**FRD2**	0	0	1	0	0	0	0	70
**FDH2**	0	0	0	0	1	0	0	330
**NADH6**	0	0	0	0	0	1	1	52
**NADTRHD**	0	0	0	0	0	1	0	0
**total**	291.25	431.25	431.25	291.25	970.75	867.5	867.5	
**ATP/cost**	0.00215	0.00145	0.00145	0.00215	0.00142	0.00202	0.00202	

Appendix 3 Table 4: Pathways used to transform each additional currency metabolite into ATP. Each reaction used is stated in left column and the cost associated with that reaction is on the right column. The relative fluxes necessary to transform 1mol of each metabolite into ATP are stated in mols. The total ATP produced by each metabolite is stated in the *ATPS4r* reaction row.

The ATP produced and cost associated with it was the added to each pathway according to each metabolite imbalance for a final pathway cost and energy production potential.

For figure 4 in the manuscript some redundancies in these pathways were removed:

- Since currency metabolites were deleted the reactions *ME1* and *ME2* became the same, resulting in two pathways that are similar aside from those reactions. Only pathways using *ME1* were considered for the figure.
- Similarly, reactions *MDH*, *MDH2* and *MDH3* became the same, and only reactions using *MDH* were considered.
- Pathways imported glucose in two ways, either through *GLCpts* or *HEX1*. Only reactions using *HEX1* were considered.

This tailoring was done to remove similar pathways and make the plot clearer. We have also produced the same plot with all 4418 pathways and the same pathways stood out.

Citations

1. Orth, J.D., Fleming, R.M.T., Palsson, B.Ø.: Reconstruction and Use of Microbial Metabolic Networks: the Core Escherichia Coli Metabolic Model as an Educational Guide. EcoSal, (2010)
2. Reed J.L., Vo T.D., Schilling C.H. and Palsson B.Ø. **An expanded genome-scale model of Escherichia coli K-12 (iJR904 GSM/GPR)**. *Genome Biol*, 2003. 4:R54.

3. Schellenberger, J., Park, J.O., Conrad, T.C., and Palsson, B.Ø., **BiGG: a Biochemical Genetic and Genomic knowledgebase of large scale metabolic reconstructions**. *BMC Bioinf*, 2010. **11**:213.
