# Supplementary material for: Predicting internal cell fluxes at sub-optimal growth
Source: BMC Syst Biol. 2015 Apr 3;9:18. doi: 10.1186/s12918-015-0153-3 (PMC4397736; doi:10.1186/s12918-015-0153-3)
Supplement: Additional file 7 — Figure S2. Cost values comparison. Cost-optimal simulations using molecular weights alone, thermodynamic penalties alone, a combination of the two and uniform costs are presented for major central carbon metabolism reactions in the range of 100 to 50% of maximal growth. [file 12918_2015_153_MOESM7_ESM.pdf]

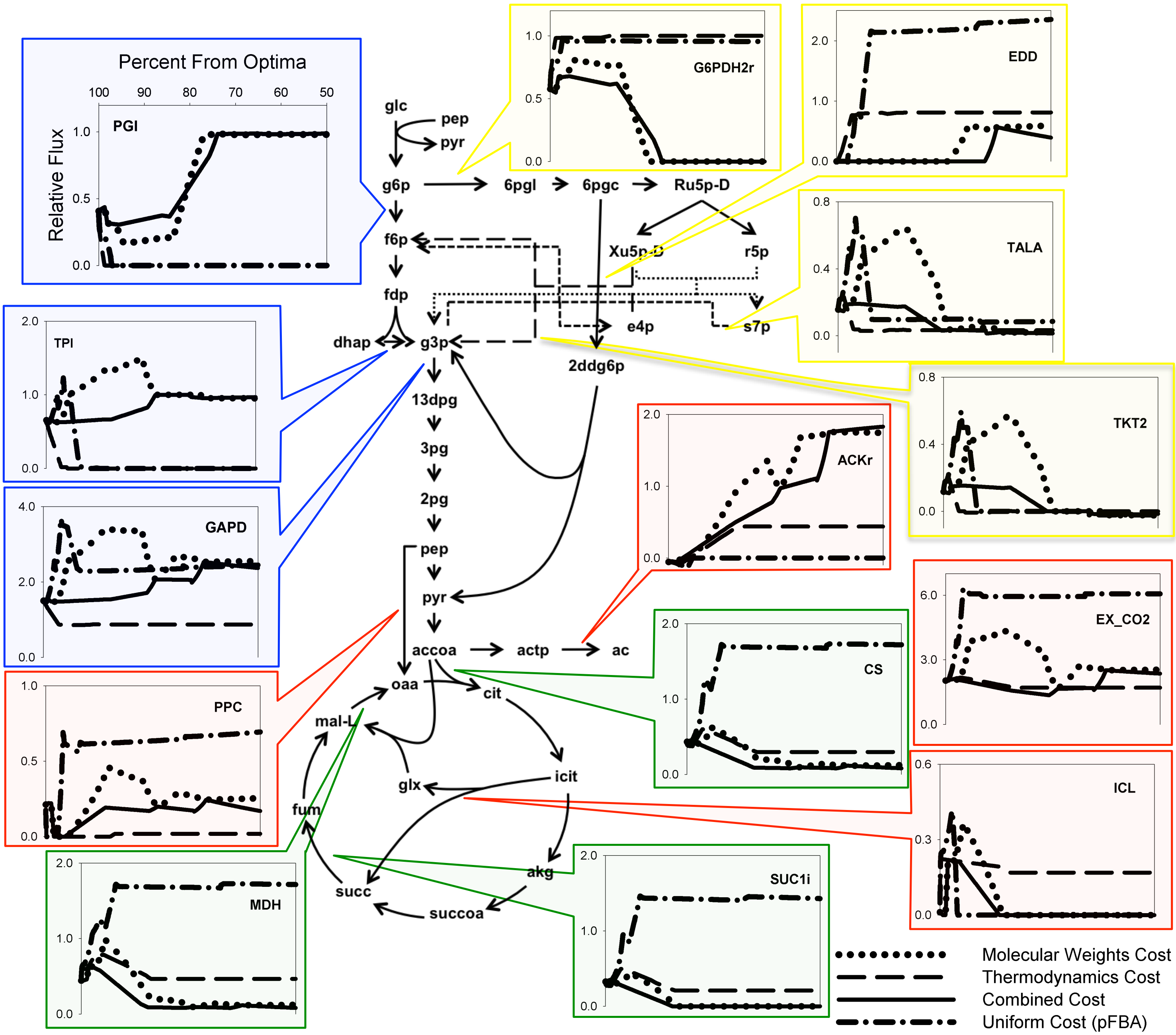

SI Figure 2: Comparison between all four costs: (1) molecular weights only, (2) thermodynamic penalty only, (3) combination of molecular weight and thermodynamic penalty and (4) uniform costs. While the implementation of molecular weights and thermodynamic values provide constantly changing metabolic profiles, the implementation of a uniform cost quickly converges to a unique metabolic profile.
