# Supplementary material for: Predicting internal cell fluxes at sub-optimal growth
Source: BMC Syst Biol. 2015 Apr 3;9:18. doi: 10.1186/s12918-015-0153-3 (PMC4397736; doi:10.1186/s12918-015-0153-3)
Supplement: Additional file 8 — Figure S3. Error and correlation calculations. Correlation and sum of squared error are calculated between all simulated and experimental flux distributions for major central carbon metabolism reactions. [file 12918_2015_153_MOESM8_ESM.pdf]

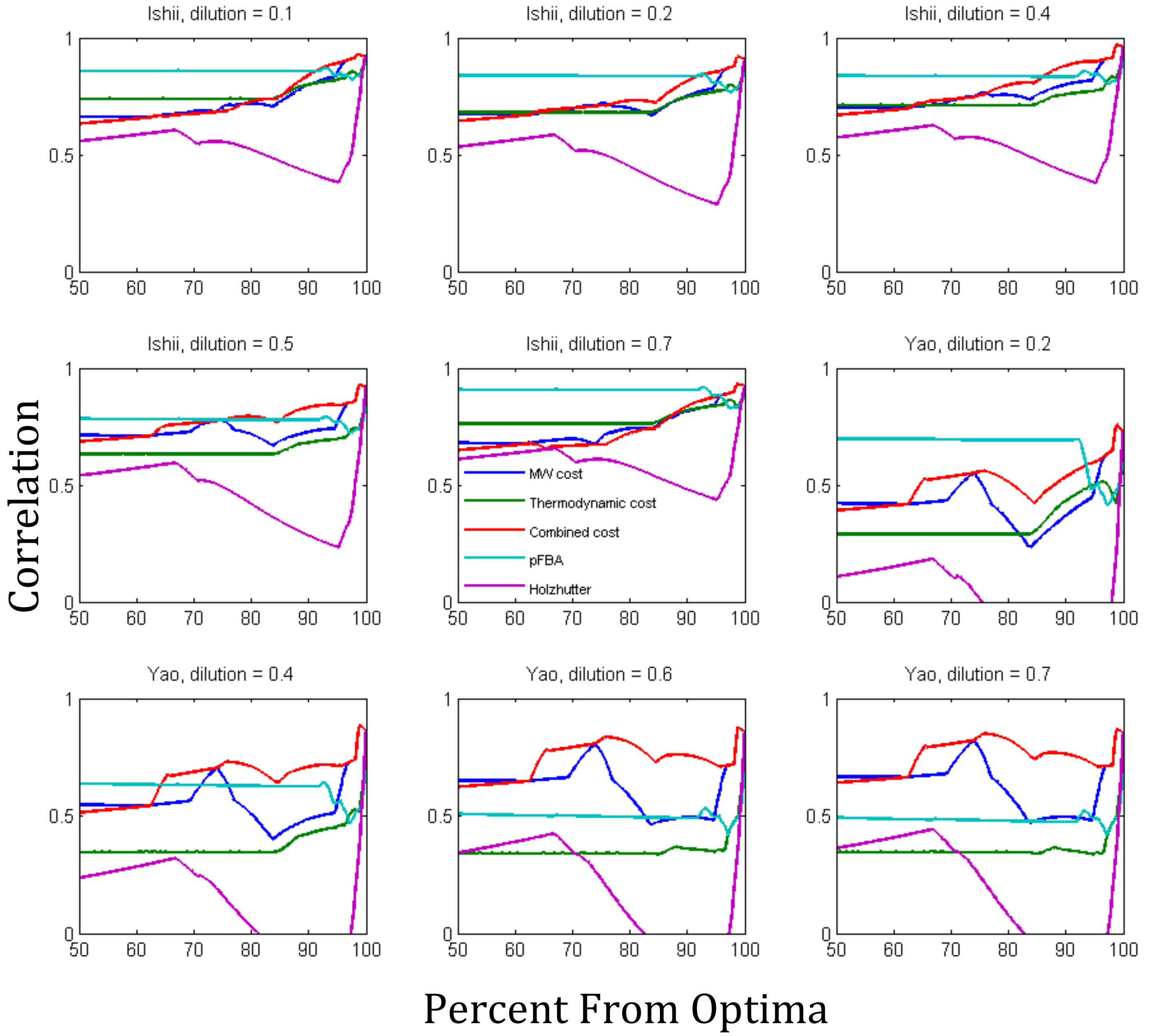

SI Figure 3 A: Correlation between simulated and experimental metabolic states between all data points and all cost-optimal simulated values ranging from 100 to 50% of optima. Each panel represents a data point and each line represent correlation values between the simulated value and the corresponding data point. All four costs used in **SI figure 2** were analyzed. Metabolic states were defined by 19 reactions for the Ishii et. al. data (*GLCpts, G6PDH2r, PGI, GND, PFK, FBA, TPI, GAPD, TKT2, TALA, CS, ICDHyr, ICL, SUCDi, MDH, PPC, EX_co2(e), ACKr* and *PYK*) and 16 reactions for the Yao et. al. data (all but *FBA, TPI* and *EX_co2(e)*).

Simulations using the combined cost function perform particularly well in the sub-optimal space for high dilution rate experimental data. *pFBA* reaches higher correlation values for the Ishii data and lower dilution values.

A similar method to that performed by Holzhütter (2004) was also included in the analysis. This method was simulated by considering only thermodynamic costs for reversible reactions in the direction opposite to the thermodynamically favorable, where the cost is higher than 1. For these simulations we used α=1. Costs for irreversible reactions and reactions in the thermodynamically favorable direction were considered to be 1. High reactions costs were rounded to a maximum of 10^5.

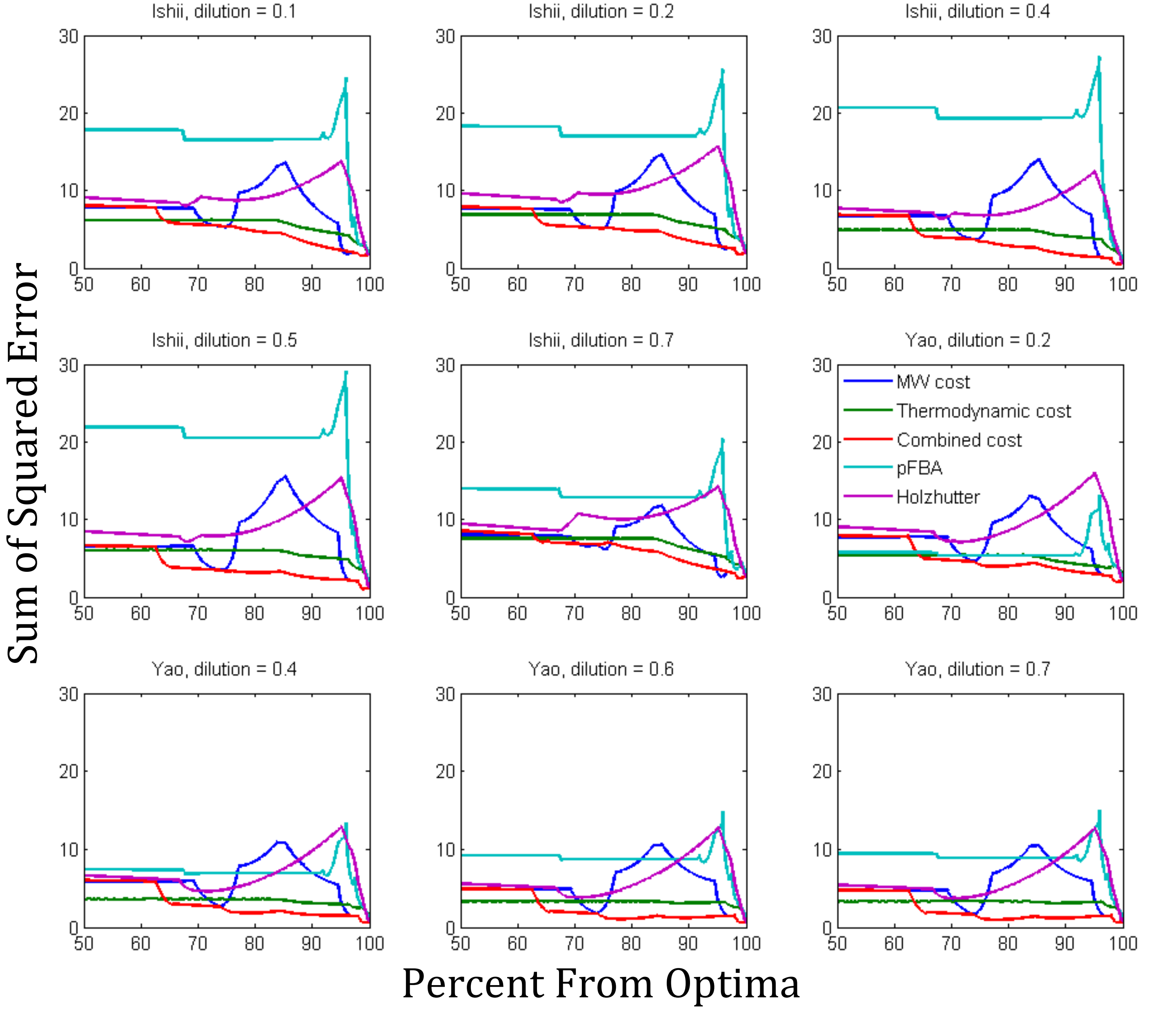

SI Figure 3 B: Sum of squared errors were calculated between the same data points and simulated fluxes as the ones in the previous figure, including the same set of reactions. We again see that simulations using the combined cost function reach the lowest error in the sub-optimal space, particularly for high growth rates. Interestingly, the *pFBA* simulated values that reach the highest correlation with the Ishii dataset, also reach the highest error.

Although the lowest error and highest correlations values are predicted by the optimal growth, here we see that the implementation of the combined cost considerably increases correlation and reduces error between data and simulated values in the sub-optimal space.
