# Supplementary material for: Predicting internal cell fluxes at sub-optimal growth
Source: BMC Syst Biol. 2015 Apr 3;9:18. doi: 10.1186/s12918-015-0153-3 (PMC4397736; doi:10.1186/s12918-015-0153-3)
Supplement: Additional file 9 — Figure S4. Error and correlation calculations for knockout strains. Correlation and sum of squared error are calculated between MFA experimental data and simulated flux distributions using the combined cost function for six knockout strains. [file 12918_2015_153_MOESM9_ESM.pdf]

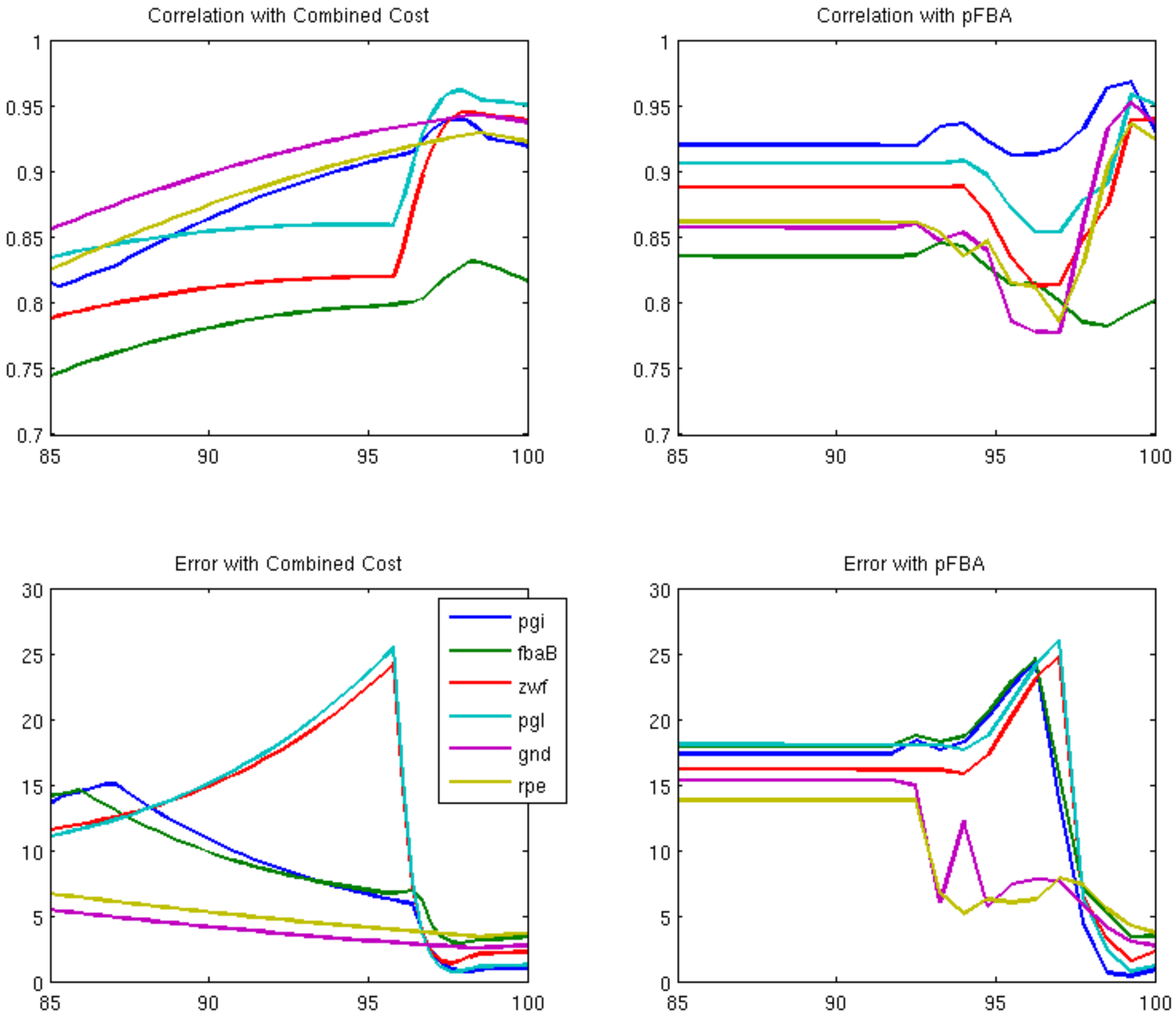

SI figure 4: Correlation and sum of squared error between simulated values and MFA experimental fluxes for six knockout strains by Ishii et. al., using both the combined and uniform costs. The same 19 reactions included in **SI figure 2** have been used in this figure. By using a combined cost, cost-optimal simulations present similar correlation and error in a sub-optimal space from 95% to maximal growth.
